# Differentiating pain-related distress from depression in people with persistent musculoskeletal pain: a mixed methods study

**DOI:** 10.1097/j.pain.0000000000003926

**Published:** 2026-03-13

**Authors:** Hollie Birkinshaw, Tamar Pincus, Stephanie Hughes, Beth Stuart, Carolyn Chew-Graham, Paul Little, Michael Moore, Noureen Shivji, Adam W.A. Geraghty

**Affiliations:** a School of Primary Care, Population Sciences and Medical Education, Primary Care Research Centre, University of Southampton, Southampton, United Kingdom; b Department of Psychology, University of Southampton, Southampton, United Kingdom; c Wolfson Institute of Population Health, Queen Mary University of London, London, United Kingdom; d School of Medicine, Keele University, Newcastle, Staffs, United Kingdom

**Keywords:** Musculoskeletal, Distress, Depression, Mixed methods, Primary care

## Abstract

Depressive symptoms are commonly experienced by people with persistent musculoskeletal (MSK) pain. There is evidence for some individuals that this may be best characterised as pain-related distress; a reaction to living with the impact of pain, rather than a depressive illness. No explorations exist of how to differentiate between these in primary care. This study aimed to explore key factors that may differentiate between pain-related distress and depression, and the effectiveness of existing symptom screening tools (Patient Health Questionnaire-9 [PHQ-9] and 4-Dimensional Symptom Questionnaire [4DSQ]) in identifying distress through a mixed methods approach. First, 21 general practitioners and 21 people with pain were interviewed about their experiences of pain-related distress. Second, 597 primary care patients with MSK pain completed a cross-sectional questionnaire study reporting on their pain, mental health symptoms, and other life events. Qualitative data suggested key factors distinguishing between pain-related distress and depression included general positive outlook, physical function, and acceptance. Quantitative findings showed that the PHQ-9 may overcategorise participants as depressed; of the 207 participants classified as distressed (but not depressed) on the 4DSQ, 118 (57%) were categorised as moderately or severely depressed on the PHQ-9. Lower positive outlook and sleep interference scores, and higher PHQ-9 and perceived stress scores significantly predicted participants experiencing distress only compared with distress and depression, matching the qualitative findings. These findings indicate that differentiation between pain-related distress and depression may be possible in primary care consultations for persistent musculoskeletal pain. This is essential to ensure that patients are offered appropriate, acceptable, and effective management.

## Abstract 2

Supplemental Digital Content is Available in the Text.

Pain-related distress may be identified in musculoskeletal pain through clinical conversations covering optimism, patient history, perceived stress, and 4DSQ questionnaire scores.

## 1. Introduction

Depressive symptoms are commonly experienced alongside persistent musculoskeletal (MSK) pain, with depression prevalence reported as high as 39%.^34,46^ However, the label of comorbid depression associated with persistent pain may mask an important distinction between “pain-related distress” and depressive disorder that co-occurs with long-term pain.^30,49^ Although both processes may overlap, the concept of pain-related distress may be more appropriate for those where the pain is functioning primarily as an interruptive stressor that people are struggling to manage. In these cases, symptoms such as sadness, poor sleep, irritation, and negative future health perceptions may be more common.^30,36,49^ For some people, pain-related distress may develop into depressive disorder or depressive disorder may be pre-existing. In these groups, symptoms associated with negative self or life evaluations such as worthlessness and meaninglessness may be more frequent, occurring *in addition* to distress-related symptoms. Identifying when a person is experiencing pain-related distress as opposed to, or alongside, depressive disorder is important; it may support more effective targeting and tailoring of treatments.

There is a lack of research regarding distinctions between pain-related distress and depression in clinical settings, particularly primary care, where the majority of musculoskeletal conditions are managed.^20^ People with pain and general practitioners (GPs) recognise and relate to the concept of pain-related distress; however, distinguishing it from depression can be difficult as symptoms can overlap and both conditions may coexist.^39^ Questionnaires are commonly used in healthcare services to assist clinician assessment and decision-making. A multitude of screening tools for the assessment of depressive symptoms exist; however, there are pervasive issues with criterion contamination; the diagnostic criteria for depression include a number of symptoms that could equally arise from pain.^31^ Existing pain scales that include mood items do not attempt to distinguish between pain-related distress and depression.

The 4-Dimensional Symptom Questionnaire (4DSQ) was developed in primary care settings and measures distress, depression, anxiety, and physical symptoms.^16,17,44^ The distinction between distress and depression potentially addresses the concerns regarding the overlap of distress related to persistent pain symptoms with depressive disorder.^1^ The 4DSQ has been shown to be acceptable, reliable, and valid with patients in pain in Switzerland^6^ and The Netherlands,^23^ yet to date no study has explored its use to identify distress in people with persistent pain in the United Kingdom.

In addition, identifying other potential distinguishing factors that can be integrated into clinical consultations is important. Low mood and pain do not exist in isolation; acknowledging the influence of factors on both pain and mood is critical in effective assessment,^7,12^ and exploring existing mental health scales' concordance with other relevant constructs may inform clinical conversations in consultations.

The aims of this mixed methods study were:

(1) To qualitatively explore key factors that differentiate between pain-related distress and depression in people with persistent musculoskeletal pain.
(2) To quantitatively explore the measurement of depression and distress in people with persistent musculoskeletal pain using the Patient Health Questionnaire-9 (PHQ-9) and the 4DSQ.
(3) To explore the concordance of the 4DSQ with other constructs potentially related to pain-related distress.

## 2. Methods

### 2.1. Study design

This study is underpinned by a pragmatic philosophical approach, which emphasises the use of multiple research methods to address complex research questions and the prioritisation of the practical application of knowledge.^14^ Specifically, it allows the integration of subjective experiences with measurable indicators, as reflected by the study aim and methods.

This study used a sequential-exploratory mixed methods design. Firstly, interviews with both GPs and people with pain were conducted and analysed (January-December 2021). This was followed by a cross-sectional survey completed by people with pain, depression, and both pain and depression (January 2022-March 2023). The design of the study purposefully facilitated the findings from the qualitative data to directly inform the measures included in the questionnaire, to enable further exploration of these concepts. Data from both studies were then synthesized together to develop conclusions, implications, and future research recommendations.

### 2.2. Patient and public involvement and engagement

A group of people living with persistent musculoskeletal pain formed the patient and public involvement and engagement (PPIE) group for this study and were involved at all stages. The group met regularly throughout the study period, and informed study design, public-facing documents, and analysis of findings. Specific amendments made after PPIE feedback include:

(1) Development of recruitment strategies for qualitative research, particularly the use of local radio.
(2) Phrasing of the qualitative topic guide to ensure relevance.
(3) The accessible layout of the questionnaire after PPIE members piloting.
(4) Inclusion of a 1 item, 0 to 10 coping item, as PPIE members felt that this topic needed to be direct and straightforward, voicing that a 0 to 10 scale was more appropriate than existing coping questionnaires (eg, Pain Coping Inventory).

### 2.3. Qualitative interviews

#### 2.3.1. Design

This study used semi-structured, iterative interviews that were conducted with people with persistent pain and distress, and GPs to explore people's experiences of distress and depression in relation to pain, and GPs' experiences of managing pain-related distress. Secondary findings from these interviews, focusing on GP management of pain in the consultation, have been published elsewhere.^39^

#### 2.3.2. Ethics

This study was approved by Southeast Scotland Research Ethics Committee (Integrated Research Application System ID: 291138) and Keele University Ethics committee (reference number: MH-200129).

#### 2.3.3. Recruitment

People with pain were recruited through both general practice electronic health records and public advertising (including advertisements in public shops, local radio, and social media). Potential participants were required to have experienced MSK pain for more than 3 months and low mood. The use of low mood as an inclusion criterion ensured that the interviews could explore the conceptualisation of this experience indepth; however, once invited no further screening procedures were undertaken. People were excluded if they were unable to consent to take part in research or were unable to complete questionnaires in English.

General practice electronic health records were searched for patients with codes for persistent musculoskeletal conditions (eg, osteoarthritis) and prescribed an antidepressant, to initially identify potential participants who may have experienced low mood in addition to their pain. Antidepressant prescriptions were used in the initial identification of potential participants to mitigate the known variation in GP coding for low mood. As antidepressants can also be prescribed for pain or sleep management for people with persistent pain, a prescription specifically for low mood was not required, and the purpose of the prescription was explored in the interviews.

Identified patients were sent an invitation letter and participant information leaflet through the post, inviting them to take part in the study by contacting the researcher. Participants recruited through public advertising were invited to contact the researcher to take part if they had musculoskeletal pain that affected their emotions (eg, feeling low, anxious, stressed, or depressed).

General practitioners were recruited through professional networks, through the Clinical Research Network, and through snowball sampling methods. Participants were recruited between April 2021 and November 2021.

#### 2.3.4. Procedure

Potential participants contacted the researchers (N.S. and H.B.) to express interest in taking part. Researchers confirmed eligibility and once participants had consented, interviews were arranged. Participants completed the Brief Pain Inventory^9^ before the interview, and their answers were used to add individualised prompts to the topic guide. All interviews were conducted by telephone or video call (eg, Microsoft Teams). Interviews were recorded, transcribed verbatim, and analysed. Between interviews, the topic guides were updated iteratively based on interim analyses and reflexive team discussions. Data collection was guided by the principle of information power.^26^ Although recognising that new insights may always be identified with additional interviews, data collection ended when the research team determined that additional interviews were unlikely to provide substantially new insights to the research aims, and that the codes identified were well supported. This was determined through regular, reflexive team meetings with T.P. (Chartered Psychologist), C.C.-G. (Academic GP), A.G. (academic psychologist), H.B. and N.S. (qualitative researchers), with input from the PPIE group. Recruitment materials and topic guides are available in the Supplementary Material, http://links.lww.com/PAIN/C455.

#### 2.3.5. Analysis

Interviews were analysed using thematic analysis using the principles of constant comparison.^4,18^ This approach focuses on meaning across the dataset of both people with pain and GPs, allowing researchers to understand collective and shared experiences. Themes were generated iteratively, through coding of the data firstly in each individual transcript, and then across the whole dataset. Coding was led by H.B. (qualitative researcher), with input from the research team.

### 2.4. Quantitative survey

#### 2.4.1. Design

A cross-sectional survey of patients from general practices in England with persistent musculoskeletal pain, depression, and persistent musculoskeletal pain and depression.

#### 2.4.2. Ethics

This study was approved by South Central—Hampshire B Research Ethics Committee (Integrated Research Application System ID: 303304).

#### 2.4.3. Recruitment

Potential participants were identified through searches of GP practice registers in 11 GP practices throughout England. Potential participants were identified through 3 searches: (1) people with persistent musculoskeletal pain without depression; (2) people with persistent musculoskeletal pain and depression; and (3) people with depression without persistent musculoskeletal pain. Patients were identified through electronic health record codes in the last 12 months. Patients were excluded from invitation if they were unable to consent to take part in research or were unable to complete questionnaires in English.

#### 2.4.4. Procedure

Eligible patients were sent a questionnaire pack through the post containing the information leaflet, questionnaire, freepost envelope, and a link to complete the questionnaire online if they wished.

#### 2.4.5. Measures

The questionnaire contained multiple scales relating to health, mood, pain, and life events. An overview of all measures is given in Table 1.

**Table 1:** Measures included in the questionnaire.

Domain	Variable	Measure
Demographics	Age	Date of birth
	Gender	Self-reported (male, female, nonbinary, prefer not to say)
	Work	Employment status and job title
	Education	Highest qualification
	Ethnicity	Self-reported (using Office for National Statistics 2021 template questions)
Health	Presence of musculoskeletal pain	Current or previous diagnoses and episodes, type of pain, pain duration
	Presence of depression	Current or previous diagnoses and episodes, duration
	Presence of anxiety	Current or previous diagnoses and episodes, duration
	Antidepressant use	Current use, purpose (pain, mood, or both), effectiveness
Mood	Depression	PHQ-9
	Distress and depression	4DSQ
	Anxiety	GAD-7
	Positive outlook	Positive Outlook Subscale of DAPOS
	Optimism	State Optimism Measure
	Susceptibility to low mood	0-10 NRS
	Stress	Perceived Stress Scale
	Life events	List of Threatening Experiences Questionnaire
Physical health	General health	SF-36 General Health subscale
	Physical function	SF-36 Physical Functioning subscale
	Physical limitations	SF-36 Physical Role Limitations due to Physical Health subscale
	Pain	SF-36 Bodily Pain subscale
MSK pain	Pain intensity	Worst, least, average, current on 0-10 NRS
	Coping with pain	0-10 NRS
	Pain interference	0-10 NRS for general activity, mood, and sleep
	Pain acceptance	Chronic Pain Acceptance Questionnaire-2 item

4DSQ, 4-Dimensional Symptom Questionnaire; DAPOS, Depression, Anxiety and Positive Outlook Scale; GAD-7, Generalised Anxiety Disorder questionnaire-7; NRS, Numerical Rating Scale; PHQ-9, Patient Health Questionnaire-9; SF-36, 36-Item Short Form Health Survey.

##### 2.4.5.1. Demographics

Age, gender, ethnicity, educational attainment, employment status, pain duration, pain site, whether currently taking antidepressants (and indication), previous diagnosis of depression or anxiety, and whether this was pre or post pain onset.

##### 2.4.5.2. Pain intensity

Four numerical rating scales covering current pain, average, worst, and least pain over the last 2 weeks on a 0 to 10 scale were used. The numerical rating scale has good psychometric properties for pain, and using the composite score increases reliability and validity.^19,50^

##### 2.4.5.3. Pain interference

Pain interference with general activity, mood, and sleep was measured on numerical rating scales from 0 to 10, with 0 as “does not interfere” and 10 as “completely interferes.” These items are based on the longer Brief Pain Inventory Interference scale, which has strong reported validity and reliability.^9^ Three items were selected to reduce burden on participants, as they refer to domains reported as having the greatest impact in our stage one qualitative study.

##### 2.4.5.4. Coping with pain

Peoples' perceptions of how they are coping with pain were measured with a single-item numerical rating scale from 0, “not coping at all” to 10, “coping extremely well,” over the last 2 weeks. This is a single-item questionnaire developed for this study alongside our PPIE group based on our qualitative research, suggesting that coping was an important factor relating to distress.

##### 2.4.5.5. Pain acceptance: Chronic Pain Acceptance Questionnaire-2

The Chronic Pain Acceptance Questionnaire-2 (CPAQ-2) is a brief 2-item measure developed from the full Chronic Pain Acceptance Questionnaire comprising of 20 items in 2 subscales (activity engagement and pain willingness). Chronic Pain Acceptance Questionnaire-2 uses 2 items rated from 0 “never true” to 6 “always true.” The CPAQ-2 has been shown to account for over 60% of the variance in the CPAQ-20.^47^

##### 2.4.5.6. Emotional distress: The Four-Dimensional Symptom Questionnaire

The 4DSQ comprises 4 subscales measuring distress, depression, anxiety, and physical symptoms (somatisation).^44^ Participants rate how often they have experienced the listed symptoms using “no” (0), “sometimes” (1), often, very often, or constantly (2). The depression and anxiety subscales have strong criterion validity with structured diagnostic interviews for depression and anxiety.^42^ The distress subscale represents a unique conceptualisation but has good criterion validity with GP psychosocial assessments.^44^ The 4DSQ has been translated into English, and the subscales have been shown to measure the same constructs as the original Dutch subscales (Cronbach alpha for the scales ranged from 0.85 to 0.92).^43^ The 4DSQ subscales can be categorised into cases of “moderately elevated” or “severely elevated.” The cut points are >10 and >20 (out of 32) for distress, >2 and >5 (out of 12) for depression, and >3 and >8 (out of 24) for anxiety.

##### 2.4.5.7. Depression: the Patient Health Questionnaire-9

The PHQ-9 is a 9-item scale that corresponds directly to the Diagnostic and Statistical Manual of Mental Disorders IV (DSM IV) criteria for Major Depressive Disorder. Participants answer items regarding depressive symptoms on a 4-point scale ranging from 0 (not at all) to 3 (nearly every day). The PHQ-9 has strong convergent validity with measures such as the Beck Depression Inventory, and internal consistency ranging from 0.86 to 0.89.^24^ The PHQ-9 categorises scores into “none” (0-4), “mild” (5-9), “moderate” (10-14), “moderately severe” (15-19), and “severe” depression (≥20).

##### 2.4.5.8. Anxiety: the Generalised Anxiety Disorder Questionnaire-7

The Generalised Anxiety Disorder Questionnaire-7 (GAD-7) is a 7-item scales that corresponds closely the DSM IV criteria for Generalised Anxiety Disorder.^40^ Participants are asked to answer questions regarding anxiety symptoms on a 4-point scale ranging from 0 (not at all) to 3 (nearly every day). The internal consistency of the GAD-7 is high (alpha = 0.92).

##### 2.4.5.9. Stress: the Perceived Stress Scale

The perceived stress scale (PSS) is a measure of “perceived stress,” specifically, the extent to which situations in a person's life are perceived as stressful. The PSS is the most widely used measure of perceived stress internationally, and psychometric study consistent report validity of >0.70 (alpha).^25^ The PSS is a 10-items scale where participants answer how often they have experienced certain feeling and thoughts on a 0 (never) to 4 (very often) scale.

##### 2.4.5.10. Physical function: SF-36 physical functioning subscale

Physical functioning was assessed using the 36-Item Short Form Health Survey (S-36) physical functioning subscale.^40^ This subscale consists of 10 items measuring ability for self-care, walking, climbing stairs or hills, bending, lifting, and moderate and vigorous activities.^48^ Scores are converted to a 0 to 100 scale, where 100 indicates the most favourable health state.

##### 2.4.5.11. Physical limitations: SF-36 role limitations because of physical health subscale

Limitations in work or other regular activities because of physical health considerations was measured by the SF-36 role limitations because of physical health subscale. It comprises of 4 questions regarding time spent on activities, limited work, and accomplishing less than desired over the previous 4 weeks.^48^ Scores are converted to a 0 to 100 scale, where 100 indicates the most favourable health state.

##### 2.4.5.12. Positive outlook

Positive outlook will be measured using the 3 positive items from the Depression, Anxiety and Positive Outlook Scale (DAPOS).^32^ Items are scored from 1 “almost never” to 5 “almost all the time.” The DAPOS has been shown to reliably measure positive outlook with strong evidence of reliability in differing samples of people with pain.^32^

##### 2.4.5.13. Optimism: the State Optimism Measure

The State Optimism Measure is a 7-item scale that designed to measure state optimism.^28^ Items are answered on a 5-point scale from strongly disagree to strongly agree; internal consistency ranges from 0.92 to 0.96.

##### 2.4.5.14. Threatening life events: List of Threatening Experiences Questionnaire

The List of Threatening Experiences Questionnaire (LTE-Q) will be used to measure stressful life events over the last year. The LTE-Q comprises 12 life events that cover major categories of life adversity including bereavement, health, relationships work, and financial problems.^5^ Participants answer yes or no over the reporting period (12 months). Internal consistency for the questionnaire is high (Cronbach alpha = 0.84).

#### 2.4.6. Sample size calculation

Sample size was calculated on the basis that analyses are primarily exploratory and descriptive. It was determined that 600 participants would provide 90% power to detect an odds ratio of 1.35 for the relationship between caseness on the existing scales and cluster, with alpha 0.05.

#### 2.4.7. Analysis

Although electronic health record codes were used as an initial method of identification, participants were grouped according to their self-report of pain or depression for analysis. This was decided as participants' self-reported pain or low mood experiences were markedly different from that indicated by the electronic health records. Of the 852 participants that returned the questionnaire, 118 reported no current pain or low mood and were excluded, and 74.7% of the participants identified through the “depression only” search also self-reported persistent musculoskeletal pain. This reflects the known inaccuracies associated with diagnostic coding in primary care^22,45^ and the inability of health systems to align with the fluctuating nature of pain. To ensure statistical power, we created a separate dataset of participants with self-reported persistent pain for analysis.

Data were analysed in Stata. Data were analysed first descriptively and secondly using a multivariable logistic regression model. The model included all measures as covariates, along with demographic variables including gender, age, education, employment status, ethnicity, and the occurrence of recent life events. This approach allowed the determination of the independent effect of each variable on the odds of being categorised in the distress and depression group vs the distress-only group, while controlling for the influence of all other factors.

## 3. Results

### 3.1. Qualitative findings

In total, 42 interviews were conducted, 21 with people with persistent musculoskeletal pain and 21 with GPs. The majority of patient participants were female (66.6%), White British (81.0%), and the average age was 55.1 years. Twelve participants (57.1%) reported a current prescription of antidepressants, with the majority (58.3%) reporting low mood as the primary reason. For GPs, most participants were male (61.9%), White British (71.4%), and the average age was 47.3 years. A full overview of demographics are provided in Table 2. Three main themes were developed: “the intrinsic link between pain and distress,” “traits of distress,” and “use of screening tools.”

**Table 2:** Qualitative participant demographics.

Characteristic	People with pain	GPs
Gender (n, %) self-disclosed		
Male	7 (33.3%)	13 (61.9%)
Female	14 (66.6%)	8 (38.1%)
Age (mean, SD)	55.1 (11.9)	47.3 (10.4)
Ethnicity (n, %)		
White British	17 (81.0%)	15 (71.4%)
Asian British	2 (9.5%)	5 (23.8%)
African British	2 (9.5%)	0 (0.0%)
Other	0 (0.0%)	1 (4.8%)
Years living with pain (mean, SD)	12.7 (7.6)	N/A
Years of experience (mean, SD)	N/A	17.6 (11.0)
Recruitment strategy (n, %)		
Media	12 (57.1%)	4 (19.0%)
Snowballing	2 (9.5%)	4 (19.0%)
Clinical Research Network	7 (33.3%)	8 (38.1%)
Professional networks	N/A	5 (23.8%)
Antidepressant use (n, %)		
Had prescription	12 (57.1%)	N/A
Reason for antidepressant (n, %)		
Pain	2 (16.6%)	N/A
Mood	7 (58.3%)	N/A
Combination	3 (25.0%)	N/A

GP, general practitioner.

#### 3.1.1. The intrinsic link between pain and distress

All participants acknowledged that low mood is a natural consequence of living with persistent pain. Distress was recognised as a separate concept to depression, with distress relating to situational contexts that could be addressed, whereas depression is a more severe condition.

*I would say, you know if they're in distress, for me that means there are situational things that can be corrected […]. Whereas depression is a very different thing completely and I think we use the term quite loosely at the moment, with people get labelled with something but actually, you know if you worked in a psychiatric ward, you'll see what true depression is or real depression is, it’s a very, very different thing. And so yes, you can feel low but that doesn't equate to sort of biological depression—*GP8

As pain itself is an unpleasant experience by design, participants considered distress is also an inherent part of that. General practitioners reported that they expected distress to accompany pain that is not resolving and that this was a normal reaction.

*I think pain and distress are conceptually very linked, aren’t they? It would be rare to find someone, well arguably it’s with the definition of pain to have pain and not be distressed*—GP11

As a result of this intrinsic link between pain and distress, one key distinguishing point identified is that if the pain could be resolved or treated, then distress also resolved. When pain is present or unmanageable, then the distress reappears.

*If my treatment works, and I have been through a lot of treatments in this, if it works I feel that I pretty much have a perfect life—*P2

*I think you can just ask and say, 'If we could wave a magic wand and take this away, how would you feel?' If they said, 'I feel absolutely fine,' you know that that is the thing—*GP9

This timeline of distress being a reaction to pain appeared key to distinguishing between pain-related distress and depression. The majority of GPs reported that they would take into consideration a patient's previous history of mental health problems, particularly before they had pain, if possible, when assessing whether they may have distress, depression, or both:

*I think there’s probably a number of elements to help guide you. I mean background, past medical history is always useful, so you can see if they’ve had previous mental health issues or they’re already on mental health medications—*GP2

A number of people with pain identified this timeline within their own history, describing their history of depression before the onset of pain. This self-reflection and identification allowed them to recognise the differences between distress and depression and explore how they interact.

*I’ve always had poor mental health; I’ve had chronic pain since my mid to late twenties. The depression was already around before then, so you have to kind of unpick what is causing the depression or exacerbating the depression—*P11

Importantly, it was recognised by both GPs and people with pain that both distress and depression can coexist; having a previous or current diagnosis of depression does not preclude experiencing pain-related distress; and both are important to recognise and address.

*I think there’s two distinct things here. I think there’s physical pain which I presume is causing distress and then there’s the depression. I think there’s a physical and a mental side to it and I think personally they’re both equally as important—*P21

*When does distress become depression you know that’s what you have to do you know and not all of the distress patients will have depression as well. But they’ll have some depressive symptoms, I think—*GP21

#### 3.1.2. Components of pain-related distress

In addition to the timeline of low mood and pain, a number of concepts were identified across the interviews that may help in differentiating between pain-related distress and depression. These were physical function, positive outlook, and pain acceptance.

##### 3.1.2.1. The impact of pain on physical function and subsequently identity

A fundamental aspect of pain-related distress was that it primarily centred on the physical impact of pain. All people living with persistent pain expressed the negative impact of being unable to fulfil their previous activities, including work, household jobs, familial, and leisure activities.

*I still am deeply distressed that I can’t just go out and have a walk when the sun is out—*P9

This impact upon physical function was often the most distressing aspect of the pain, as the disruption in the ability to undertake usual tasks disrupted their identity.

*Yeah it’s affected my well-being in that respect but also physically as well, not being able to do what I want to do. I used to be a runner and I can’t run any more and that really has devastated me—*P3

“Frustration” was a key descriptor that was often used by both people with pain and GPs in relation to this impact on activity and identity. Again, this was used as a distinguishing factor from depression; although depression influences every part of a person's life, distress is related to the impact of pain.

*It stops me from doing things and I feel extremely frustrated, so frustrated. I will admit— I wouldn't call it depression but some days, I could cry with it. I really could—*P13

*I think if somebody is truly depressed, they are never going to feel better because it's pervasive and it's affecting every single aspect of their life; whereas, if it's secondary to a distinct pathology, I think most people can probably recognise that and it's more of a frustration perhaps—*GP9

Furthermore, GPs noted that patients experiencing pain-related distress tended to consult primarily regarding the physical impact of pain unprompted, further solidifying the link between physical activity and distress.

*So normally I would just say ‘describe what your day is like for you, or tell me about a bad day’, and I find that patients with chronic pain just automatically link in, you know ‘I was having a bad day with the pain’ or ‘I wasn’t able to do this because of the pain’, so just organically they bring it in themselves, so that’s definitely a key thing in terms of what they say—*GP16

##### 3.1.2.2. Positivity outside of pain

Although the impact of persistent pain is upsetting, positivity, particularly in relation to optimism was a recurring theme. When discussing distress, some people with pain could see positivity in areas of their lives where the pain had less of an effect, reinforcing a causal link between pain and distress.

*So, I tend to be very optimistic, I keep trying to keep cheerful for all my family members, it’s just that when I have pain I can’t do anything—*P2

This positivity was often described as a personality trait, a part of their identity that was not changed, despite the frustration experienced as a result of pain.

*Not really depression, I’m not a depressive sort of person, I’m generally quite optimistic and cheerful. But now and again it makes me fed up and cross but I wouldn’t say depressed—*P1

One GP explained that an effect of maintaining positive outlook and optimism is that pain-related distress is “compartmentalised” to the areas of their life that pain is affecting.

*I can see that it gets people down and it gets people down very quickly but they can probably compartmentalise it and say, 'I used to like hill walking and I used like playing badminton and those aspects of my life are missing.' There's a sense of loss but it can be perhaps externalised a little bit and perhaps separated from the other aspects of their life that are working—*GP9

This was highlighted as a crucial differentiator between distress and depression throughout interviews. With distress, positivity could be found in other areas of a person's life, whereas depression was ubiquitous. People with depression and pain had no areas of positivity they could identify, even when unrelated to pain.

*It’s important to look ahead and see whether there are things in the patient’s life that might improve things for them. So, they might be optimistic because they’re about to start a new job and the new job will help or they might be optimistic that the weather’s going to improve after Christmas and that will help. There might be things coming up, they might have a daughter’s wedding or something. […] If they can see no way out of it, if they’ve got that pervasive low mood, pessimism and they’re starting to feel suicidal then obviously that’s an important distinction*—GP20

##### 3.1.2.3. Pain acceptance, coping, and life adjustments

Throughout the interviews, a link between acceptance and pain-related distress was identified. Participants described that accepting that the pain was chronic and could not be cured allowed was adaptive; their focus could then turn to engaging in adaptive coping strategies and living better with pain.

*It’s a very challenging thing to accept [that the pain will never resolve] but it was also accurate and so although it was quite difficult at the time and took some adjusting to, actually it was ultimately helpful because what it allowed me to do was stop pouring my time and energy into trying to become pain free. And instead focus my time and energy on what is the best way to do things given that when you do things they cause you pain—*P11

Adopting coping strategies that facilitated continuation of everyday life was in turn directly linked to reducing distress.

*I think I've become mentally stronger. I just try and do what I can, like I've said before, within my own limitations. Everybody changes, don't they? It's a hard question to think about how I've changed but I've just coped with it as best I can. I've developed coping strategies—*P14

In contrast to acceptance, resignation was identified as an indicator of more pervasive hopelessness that is characterised in depression, with people resigning themselves to living with pain rather than focusing on adaptive coping strategies.

*Depression is more likely where somebody feels a bit resigned maybe and maybe quieter about things*

*I don’t really have any other strategies, yeah, I just kind of… because I’m so resigned to it now so it’s like… it is literally me saying come on, this is it, there’s nothing you can do—*P3

#### 3.1.3. Identifying types of low mood in consultations for pain

General practitioners discussed their processes for the assessment and identification of low mood in people with pain. Screening tools were often mentioned as a way for GPs to identify low mood in time-pressured consultations, with validated tools providing reassurance in making a diagnosis. The PHQ-9 was often referenced as being used as a diagnostic indicator of depression.

*So, with depression, I screen them. So, I like to use screening tools, so PHQ-9. So, for me, I like to have some kind of quantification of it from a depression perspective. So that’s what I like to use more than anything—*GP1

Although previous discussions had highlighted the inherent link between persistent pain and low mood, multiple GPs explained that their use of the PHQ-9 helped to differentiate between the effects of the pain and standalone low mood.

*Using scales to assess mood. So, I would often use a PHQ-9 questionnaire or an anxiety questionnaire like GAD because that would help me tease out the chronic pain on its own from the psychological and social side—*GP17

However, confidence in the PHQ-9 as a diagnostic tool was not shared by all GPs. Some expressed reservations about the appropriateness of its use in this population, expressing concerns that it may misdiagnose people as depressed based on pain and distress symptoms.

*Sure, I mean depression has got a clinical tool you know, PHQ-9, although again if you see the PHQ-9 scales it will tick most of the boxes for patients with pain related distress. It asks about sleep, it asks about mood, it asks about motivation, I think patients will not have motivation to do anything when they’re themselves in distress—*GP6

Rather, engaging in clinical conversations that include elements of the PHQ-9, with scope to discuss symptoms and ascertain context, was preferred.

*Yeah, I don't really like the questionnaires that we're all supposed to use. I think a conversation would be more organic than that but if I was really worried about somebody, I'd ask, 'What is your sleep like?' or 'Do you still enjoy anything?' That would probably be one I would ask, definitely. I would ask about appetite and relationships with other people—*GP9

These conversations allowed GPs to explore low mood symptoms in more depth to understand the complexity of the situation and identify potential factors to distinguish distress from depression.

*Okay, so the content of the conversation is different with depression. So, there’s more of a hopelessness and they’ve been in this situation a long time, they can’t see a way out. The emotionally distressed people are more agitated and a bit more aggressive really, I find. But sometimes you need to unpick and go quite deep into the conversation. You often find that people who come off as being distressed are actually underneath everything quite depressed as well—*GP10

This also allowed GPs to explore the more psychiatric aspects of depression, particularly suicidal ideation, which was used as a clear marker for depression.

*If they can see no way out of it, if they’ve got that pervasive low mood, pessimism and they’re starting to feel suicidal then obviously that’s an important distinction—*GP20

#### 3.1.4. Qualitative summary

In summary, participants universally recognised that persistent pain is inherently linked to distress. Pain-related distress was perceived as a consequence of the pain and could be alleviated if pain was resolved, whereas depression is pervasive. Key components of pain-related distress included its impact on physical function, daily activities and function; the loss of their previous roles was a primary source of distress. However, in relation to distress, people could maintain positivity in areas of life unaffected by pain, a key factor in differentiating from depression. Acceptance of pain, along with effective coping strategies, played a critical role in reducing distress. Furthermore, a large proportion of GPs discussed using the PHQ-9 to assess low mood; however, some limitations were identified and the importance of an open discussion with patients was highlighted.

**Figure 1.**
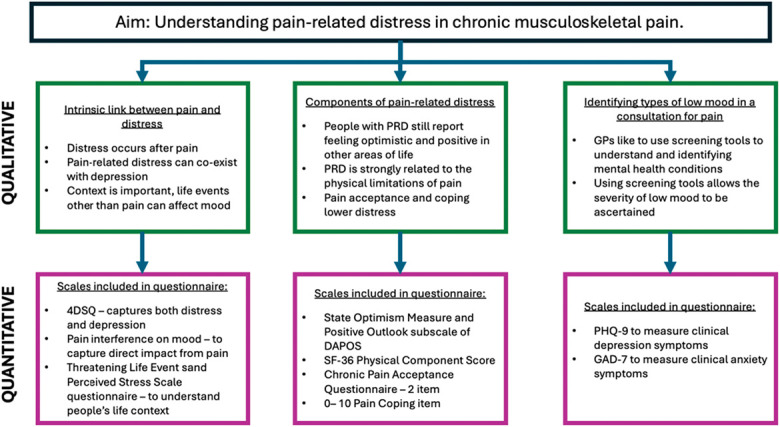
Overview of mixed methods integration.

These themes were used as the foundation for the choice of scales included in the quantitative study, as displayed in Figure 1.

### 3.2. Quantitative results

#### 3.2.1. Sample

Eleven GP practices in England mailed out invitations to a total of 5702 patients who met the inclusion criteria. In total, 852 people completed and returned the questionnaire. Of these, 597 reported persistent pain and are subsequently included in the following analyses. Participants were predominantly female, retired, and White. Full demographics are displayed in Table 3.

**Table 3:** Quantitative participant demographics.

Demographic	Proportion, n = 597
Female	415 (70.7%)
Age (mean, SD)	61.1 (15.97)
Employment	
Full-time	126 (21.5%)
Part-time	91 (15.6%)
Volunteer	9 (1.5%)
Retired	268 (45.8%)
Unemployed	91 (15.6%)
Education (highest attained)	
High school	144 (25.6%)
College	106 (18.9%)
Undergraduate	110 (19.6%)
Postgraduate	71 (12.6%)
Doctorate	20 (3.6%)
Other	111 (19.8%)
Ethnicity	
White	569 (96.4%)
Mixed	8 (1.4%)
Asian	6 (1.0%)
Black	3 (0.5%)
Other	4 (0.7%)

#### 3.2.2. Pain characteristics

The majority of participants reported experiencing musculoskeletal pain for over 1 year, with moderate pain intensity and interference (Table 4).

**Table 4:** Pain characteristics of the quantitative participants.

Demographic	Statistic
Average pain intensity	5.3 (2.18)
Duration	
3-6 mo	21 (3.6%)
7-12 mo	26 (6.2%)
1-2 y	73 (12.5%)
3-5 y	111 (19.1%)
6-10 y	123 (21.1%)
10+ y	218 (37.5%)
Interference	
General activity	5.1 (2.72)
Mood	4.5 (3.00)
Sleep	4.9 (3.09)

#### 3.2.3. Psychosocial characteristics

Participants' scores on the scales were examined to determine the average levels in the sample. Statistics are presented in Table 5. Overall, both the 4DSQ and PHQ-9 indicated that the sample reported moderate levels of depression (4DSQ cut-point >2; sample mean = 3.1 [3.88]; PHQ-9 cut-points 10-14; sample mean = 10.2 [7.47]), and anxiety (4DSQ cut point >3; sample mean = 4.9 [6.1]). On average, the 4DSQ showed that the sample was moderately distressed (4DSQ cut point >10; sample mean = 15.0 [9.40]) yet reported scores of positive outlook above the midpoint of the DAPOS subscale (mean = 9.7 [3.13]). For pain factors, the sample showed high levels of chronic pain acceptance (mean = 8.5 [2.09]) and an average score in the upper-middle range on pain coping (mean = 6.2 [2.53]).

**Table 5:** Psychosocial characteristics of participants in quantitative study.

Domain	Scale (min-max score)	Statistic*
Depression	PHQ-9 (0-27)	10.2 (7.47)
	4DSQ depression (0-12)	3.1 (3.88)
	Self-reported “yes”	346 (59.0%)
Anxiety	GAD-7 (0-21)	6.3 (5.87)
	4DSQ anxiety (0-24)	4.9 (6.11)
	Self-reported “yes”	285 (48.6%)
Distress	4DSQ distress (0-32)	15.0 (9.40)
Positive outlook	DAPOS (3-15)	9.7 (3.13)
Optimism	SOM (0-35)	19.1 (7.36)
Stress	PSS (0-40)	18.7 (8.44)
Physical function	SF-36 physical functioning (0-100)	41.9 (30.85)
Physical limitations	SF-36 role limitations because of physical health (0-100)	27.0 (37.2)
Chronic pain acceptance	CPAQ-2 (0-12)	8.5 (2.09)
Pain coping	0-10 NRS (0-10)	6.2 (2.53)

* Continuous variables presented as mean and SD; categorical variables presented as n and %.

4DSQ, 4-Dimensional Symptom Questionnaire; CPAQ-2, Chronic Pain Acceptance Questionnaire-2; DAPOS, Depression, Anxiety and Positive Outlook Scale; GAD-7, Generalised Anxiety Disorder questionnaire-7; PHQ-9, Patient Health Questionnaire-9; PSS, Perceived Stress Scale; SOM, State Optimism Measure.

#### 3.2.4. Patient Health Questionnaire-9 and 4-Dimensional Symptom Questionnaire Caseness

Patient Health Questionnaire-9 and 4DSQ caseness results are displayed in Table 6. In total, the PHQ-9 classified almost half of the sample as “moderately or severely depressed” (46.9%). In comparison, the 4DSQ classified over a third of the sample as meeting the criteria for “distress only” (37.2%) and almost a quarter as “distress and depression” (23.3%). Only 1% of the sample was classified as “depression only” by the 4DSQ, suggesting that although distress can exist independently of depression, depression does not exist independently of distress.

**Table 6:** Proportion of participants as cases of the Patient Health Questionnaire-9 and 4-Dimensional Symptom Questionnaire.

Scale	Proportion
PHQ-9	
None/mild (0-9)	304 (53.1%)
Moderate/severe (≥10)	268 (46.9%)
4DSQ	
Distress only	213 (37.2%)
Depression only	6 (1.1%)
Distress and depression	133 (23.3%)
None	220 (38.5%)

4DSQ, 4-Dimensional Symptom Questionnaire; PHQ-9, Patient Health Questionnaire-9.

Further analysis explored the proportion of participants fitting into the cases of both the PHQ-9 and the 4DSQ (Table 7). Of the 207 participants classified as “distress only” by the 4DSQ, the PHQ-9 categorised over half of these as “moderately or severely depressed” (57.0%). In comparison, very strong agreement is seen in the “distress and depression” 4DSQ sample; the PHQ-9 categorised 98.5% of these as “moderately or severely depressed.”

**Table 7:** Crosstabs analysis of cases across the 4-Dimensional Symptom Questionnaire and Patient Health Questionnaire-9.

	4DSQ distress (n = 207)	4DSQ distress + depression (n = 132)
PHQ none/mild	89 (43.0%)	2 (1.5%)
PHQ moderate/severe	118 (57.0%)	130 (98.5%)

4DSQ, 4-Dimensional Symptom Questionnaire; PHQ, Patient Health Questionnaire.

#### 3.2.5. Differentiating between distress and depression

Differences in psychosocial variables between participants with pain classified as “distress only” or “distress and depression” were explored descriptively and through logistic regression. Results are displayed in Table 8. In a logistic regression model, higher PHQ-9 (odds ratio [OR] 1.23; 95% confidence interval [CI] 1.13-1.33) and perceived stress scores (OR 1.13; 95% CI 1.04-1.22) and lower positive outlook (OR 0.83; 95% CI 0.70-0.98) and interference with sleep scores (OR 0.85; 95% CI 0.73-1.00) were the variables that independently predicted distress and depression compared with distress only.

**Table 8:** Results from logistic regression analyses.

Scale	Distress only (n = 212) (Mean, SD)	Distress and depression (n = 132) (Mean, SD)	Odds ratio (95% CI)
Pain intensity	5.2 (2.56)	6.5 (2.09)	1.25 (0.97-1.61)
Pain interference—general activity	5.1 (2.56)	6.5 (2.59)	0.94 (0.75-1.18)
Pain interference—mood	4.8 (2.53)	7.0 (2.43)	1.14 (0.92-1.43)
Pain interference—sleep	5.4 (2.78)	6.5 (2.82)	0.85 (0.73-1.00)*
PHQ-9 total	11.3 (5.11)	19.2 (4.53)	1.23 (1.13-1.33)*
GAD-7 total	6.9 (4.30)	12.7 (5.20)	1.04 (0.96-1.14)
DAPOS—positive outlook	9.4 (2.34)	6.7 (2.25)	0.83 (0.70-0.98)*
State optimism measure	18.1 (5.68)	12.6 (5.01)	0.99 (0.92-1.06)
Perceived stress scale	20.6 (5.62)	27.6 (5.0)	1.13 (1.04-1.22)*
CPAQ-2	8.6 (2.19)	8.1 (2.01)	0.96 (0.81-1.14)
Pain coping	6.3 (2.34)	4.5 (2.53)	0.94 (0.75-1.18)
SF-36 physical functioning	40.3 (30.32)	30.4 (28.54)	1.01 (0.99-1.02)
SF-36 physical limitations	20.5 (32.18)	8.8 (22.56)	1.00 (0.99-1.02)

The model included all measures as covariates, along with demographic variables including gender, age, education, employment status, ethnicity, and the occurrence of recent life events. * Indicates a statistically significant result.

CPAQ-2, Chronic Pain Acceptance Questionnaire-2; DAPOS, Depression, Anxiety and Positive Outlook Scale; GAD-7, Generalised Anxiety Disorder questionnaire-7; PHQ-9, Patient Health Questionnaire-9.

## 4. Discussion

### 4.1. Conceptual and empirical differences between pain-related distress and depression

The findings support the conceptualisation of pain-related distress in people with persistent musculoskeletal pain, and its distinction from clinical depression. Identifying pain-related distress and distinguishing it from depression in clinical situations is, however, challenging. General practitioners discussed the overlap between pain-related distress and depression and the lack of any validated assessment tools for distress, primarily relying on the PHQ-9 for identifying the severity of depression. Exploring primary care patients' symptom profiles with the 4DSQ showed that this questionnaire may be useful in distinguishing between distress and depression in those with pain, when combined with sensitive and comprehensive clinical discussions. The synthesis of findings from both the qualitative and quantitative data suggest that distress is an inherent part of depression in people with persistent MSK pain but can also be experienced alone, matching previous research in the area,^39,49^ and in the wider mental health field.^13,16,17^ This suggests appropriate treatment strategies should differ.

Qualitative analysis identified several constructs that may be useful in distinguishing pain-related distress from depression including physical function, positive outlook and optimism, life context, and chronic pain acceptance and coping. When analysed quantitatively, higher levels of stress and lower levels of positive outlook and sleep interference significantly predicted depression and distress cases compared to distress only.

The significance of positive outlook as a differentiator between distress and depression aligns with the qualitative findings; people with pain-related distress reported holding positive future expectations outside of situations relating to their pain, for example, an offspring's wedding or starting a new job. This supports previous literature which shows that for future thinking tasks, people with pain and low mood tend to focus on negative health-related future events, significantly more so than people with pain but no low mood.^36^ By contrast, positive outlook is an antithesis to depression, which is characterised by pervasive hopelessness and pessimism not only about current events but also about the future, which is perceived as threatening and empty of hope.^8^ Multiple GPs referenced positivity as an indicator that could be identified in clinical conversations during usual consultations. When discussed by people with pain, their optimism and positivity tended to be characterised as a trait; often describing themselves as “cheerful” or “optimistic” people. Potentially, the positive outlook subscale of the DAPOS tapped into this characteristic moreso than the State Optimism Measure, which focused on optimism in the moment. Untangling whether positive outlook is trait-led or a state experience, and therefore potentially modifiable through clinical encounters and interventions, is a priority for future research. This is particularly important as dispositional (“trait”) optimism is positively associated with wellbeing in people with long-term health conditions, including pain,^2,3^ and specifically the lessening of psychological distress in people with persistent MSK pain.^51^

Higher levels of perceived stress were predictive of belonging to the distress + depression population rather than the distress only population. The Perceived Stress Scale measures general perceptions, linking with the conceptualisation that while pain-related distress is specifically linked to the experience of pain, depression is a complex phenomenon that may be influenced by concurrent life events unrelated to pain.^21,41^ Previous research has highlighted the relationship between perceived stress and depression,^11^ linking to the theory that the perception of stress is a symptom of depression, such that people with depression are prone to negative perceptions of stress.^25^

While the Physical Component Score of the SF-36 was not quite a significant predictor between distress and distress + depression populations, the frustration of being limited physically was strongly reiterated throughout interviews. This adds to previous research, people described losing the ability to undertake valued activities that formed a core part of their identity, including household, work, and leisure activities.^15^ This a crucial qualitative differentiation between distress and depression; participants with pain-related distress were still motivated to engage in activities but were frustrated as their pain physically restricted them. In comparison, people with depression tend to withdraw from activities not because of physical constraints, but primarily because of loss of motivation and anhedonia.^8^ Indeed, pain-related distress has been hypothesized to be part of a cyclical relationship with physical function, characterised by the suffering associated with disability.^30,37^ It may be more appropriate for physical function to be explored through clinical conversations rather than a questionnaire.

Although pain acceptance and coping were highlighted by the qualitative analysis as important constructs in relation to distress, these was not corroborated through the quantitative analysis. The choice of short tools to measure these factors (CPAQ-2 and a standalone 0-10 NRS for coping) may not have had the depth to fully capture the complexity or understanding of these factors identified in the qualitative literature, or it may be that these constructs are more appropriately elicited through conversations that measures.

Our findings also suggested that the PHQ-9 lacks sensitivity to differentiate between distress and depression and overclassifies distressed people as depressed. The almost perfect concordance between the 4DSQ and the PHQ-9 on people who fall into the “distressed and depressed” category on the 4DSQ indicates that the PHQ-9 is measuring a combination of distress and depression, without the specificity to separate the concepts. Previous research has highlighted concerns about the appropriateness of using the PHQ-9 in persistent pain populations.^1^ Cognitive interviews with people with persistent MSK pain revealed that only 3 items of the PHQ-9 are congruent with the intention to measure clinical depression; for the other items, over 50% of positive responses were attributed to pain-related or completely unrelated issues.^1^ The Hospital Anxiety and Depression Scale has been posited to be a better screening of pain-related distress than tools focusing on the DSM criteria because of being closely related to pain-related disability,^38^ yet the removal of the severe psychopathological symptoms (such as suicidal ideation) lessens its usefulness in primary care, where it is important to identify potential risk. These issues may be attenuated in the 4DSQ because of its 4 separate subscales, including a physical symptom subscale.

### 4.2. Clinical implications

The distinction between pain-related distress and depression has several implications for clinical practice. First, although the PHQ-9 is the service standard for mental health assessment in the National Health Service in the UK,^29^ it overclassifies distress as depression in people with pain and may subsequently lead patients to unnecessary and potentially suboptimal pathways. The results from this study suggest that the 4DSQ may be a more appropriate tool; it is able to indicate when a person is likely to be distressed, and despite its length is evidenced to be acceptable to patients.^6,16,23^ In addition, the inclusion of a physical symptom subscale may address the issue of criterion contamination with pain symptoms, as raised previously.^1^ Second, the importance of the clinical conversation is highlighted. Although distinguishing between distress and depression is complex, this study has identified several indicators for pain-related distress vs depression, specifically positive outlook, stress, physical function, and acceptance. A framework for GPs for primary care consultations with people with persistent MSK pain has been published.^33^ Third, the identification of optimism, positive outlook, and acceptance as key factors for pain-related distress opens the door for new types of treatment strategies for people with persistent musculoskeletal pain and distress, adjusting their goals to re-engage with valued activities.^35^

### 4.3. Advantages and limitations of the methodology

The use of mixed methods in this study is a strength as health care, pain, and distress are inherently complex and multifaceted.^33^ Mixed methods allow a rich exploration and understanding of phenomena while offering corroboration with the pragmatic processes occurring in everyday life.^10^ However, although the cross-sectional questionnaire was pragmatically suited to this study, the lack of longitudinal analysis precludes cause and effect conclusions to be drawn, and insight into how distress in the context of pain may change over time parallel to pain trajectories is lacking. Undertaking longitudinal research to explore this is a priority. In addition, future research should undertake analysis to explore whether certain scale items can discriminate between distress and depression, and whether there is potential for the development of a specific pain-related distress scale, because this was not the original focus of the 4DSQ's development. The samples in both studies, but particularly the quantitative study, are limited in their representativeness of the general population. Most survey respondents were White, female, and retired. Therefore, this study omits intersectional issues related to the experience of pain, distress, and healthcare consultations, such as structural or social challenges related to race and culture.^27^ This study also adopted a conceptualisation of pain-related distress that may not encapsulate all dimensions of psychological distress; future research in other populations exploring the conceptualisation and measurement of pain-related distress, and the utility of the 4DSQ, is essential.

### 4.4. Summary

This study suggests that distress can be distinguished from depression in people with persistent musculoskeletal pain. Our findings indicate that an existing questionnaire, the 4DSQ, may help identification of pain-related distress as it provides scores that separate distress from depression. In addition, there are several key factors that could be considered in clinical conversations to explore whether distress, depression, or both are present. Continued research on pain-related distress is important; effective integration of the concept into clinical practice can support more acceptable and appropriate care.

## Conflict of interest statement

The authors have no conflict of interest to declare.

## Supplementary Material

## Appendix A. Supplemental digital content

Supplemental digital content associated with this article can be found online at http://links.lww.com/PAIN/C455.
