# Supplementary material for: Differentiating pain-related distress from depression in people with persistent musculoskeletal pain: a mixed methods study
Source: Pain. 2026 Mar 13;167(6):1417–29. doi: 10.1097/j.pain.0000000000003926 (PMC13225238; doi:10.1097/j.pain.0000000000003926)
Supplement: Supplementary file 1 [file jop-167-1417-s001.pdf]

Differentiating pain-related distress from depression in people with persistent musculoskeletal pain: a mixed methods study

Supplementary digital content
v1.0 30th May 2025

Table of Contents

Qualitative patient invitation letter..........2

Qualitative media advert text..........5

Qualitative patient participant information leaflet..........6

Qualitative topic guide for people with pain..........10

Qualitative GP recruitment email..........12

Qualitative GP information leaflet..........15

Qualitative GP topic guide..........19

Qualitative table of themes..........22

Quantitative cover letter..........25

Quantitative patient information leaflet..........27

Quantitative cross-sectional survey..........31

Qualitative patient invitation letter

PRACTICE HEADED NOTEPAPER

De-STRESS
pain

Patient details   Date

Dear XX

We are writing to inform you that this practice is participating in a study, funded by Versus Arthritis, and working with researchers at Keele University on a study ***Developing a patient-centred evidence-based intervention to address distress in the context of chronic musculoskeletal pain.***

Your GP has identified that you have experience of living with chronic musculoskeletal pain. The researchers would like to interview patients, like you, because they want to find out what you think, they are interested in your views. There are no right or wrong answers.

The first part of the study involves participating in an interview with a researcher from Keele University. This interview will last up to 45minutes and can take place face to face (in a location convenient to you), over the telephone, or using an alternative platform such as google 'hangouts'.

If you would like to take part, please reply to the research team either by e-mail or telephone or by completing the enclosed reply form and return it in the enclosed stamped addressed envelope. A member of the study team will then contact you and send you some more information about the study. We would also be grateful if you would complete the enclosed questionnaire, and return this in the stamped addressed envelope.

Your participation is voluntary and it is up to you whether you take part. We can assure you that whether or not you take part in the interview, your healthcare will not be affected in any way, now or in the future.

Your views are very important to the researchers. Enclosed is an information leaflet which explains the study in more detail.

If you have any questions about this research, please contact the xxxxxxx directly on 01782 xxxxxxx

Thank you for considering participating in this study. If you do not wish to take part, this will not affect the care you receive from the practice in any way.

Yours sincerely

GP name

Invitation V1.0 28may2020

De-STRESS *pain*

INTERVIEW REPLY FORM

I (insert name)………………………………………………………………………… am willing to be contacted by the research term to discuss participating in the research study:
Developing a patient-centred evidence-based intervention to address distress in the context of chronic musculoskeletal pain.

Initial box to confirm

I have read the xxxxxx Interview Study Participant Information leaflet (version xx, dated xxxxxxxx) and would like to take part in an interview. ☐

I agree that a researcher can contact me to arrange the interview. ☐

The best time to contact me by telephone is (please cross all that apply):

9am – 1pm ☐ 1pm-5pm ☐

The best days to contact me by telephone are (please cross all that apply):

Monday ☐ Tuesday ☐ Wednesday ☐ Thursday ☐ Friday ☐

Please enter your contact details below:

Name…………………………………………………………………………………………………

Telephone:

Home: ……. ………………………………………**Mobile:** ……………………………………………….
(including area code)

Email ………………………………………………………………………………………

Signature………………………………… Today's date D D M M M Y Y Y Y

E.g 10 Mar 2020 for 10th March 2020

Invitation V1.0 28may2020

Please return your completed form in the envelope provided.

xxxxxxx Study Researcher
Keele University
Staffordshire
ST5 5BG

If you have any questions please ring the xxxxx Study Researcher on 01782 xxxxxx

Qualitative media advert text

Developing a patient-focussed programme to address distress associate with chronic MSK pain.

Do you have long-standing musculoskeletal pain?

Would you agree to take part in research which will help us understand how you deal with emotions, such as feeling low, anxious, stressed or depressed?

What is involved?
A single interview between 30 and 45 minutes, at a time and place convenient to you.
You will be offered a £20 voucher for your time as well as reasonable travel expenses.
How will your interview be used?
Your interview will be completely confidential and will write it up and you will not be able to identified.
Your contribution could improve the care of people with chronic pain in the future.
Are you interested in participating?
For more details, please contact
Name of researcher
E-mail:
Telephone:

De-STRESS
pain

Qualitative patient participant information leaflet

De-STRESS
pain

Information leaflet for people with pain

Developing a patient-focused programme to address distress associated with chronic MSK pain

We invite you to take part in our research study entitled "Developing a patient-centred evidence-based intervention to address distress in the context of chronic musculoskeletal pain". Before you decide whether or not you wish to take part, it is important for you to understand why this research is being done and what it will involve.

Please take your time to read this information sheet carefully. Should you have any questions please contact the principal investigator Professor Carolyn Chew-Graham (01782 734 717 or c.a.chew-graham@keele.ac.uk)

Summary:

- This study is being carried out by researchers at Keele University, Royal Holloway (University of London) and University of Southampton
- Your participation is voluntary
- The research has been received favourable ethical approval from the South East Scotland Research Ethics Committee
- Participation involves completing a short questionnaire and a single interview, lasting around 45 minutes, at a location convenient for you
- Your consent to take part will be recorded prior to the interview
- Your personal information will be made anonymous so that what you say in the interview cannot be linked back to yourself

Who is carrying out this research?

This study is being carried out by Professor Carolyn Chew-Graham at the School of Primary, Community and Social Care, Keele University, along with researchers from the Universities of Southampton and London. The study is funded by Versus Arthritis (grant number 22454).

What is the reason for this research?

People with long-standing, or 'chronic,' pain can be upset or distressed, and it is thought these feelings are different to 'clinical depression'. Thus, treatments to help the person manage that distress needs to take account of these special circumstances and the presence of pain. The aim of this study is to explore these feelings of upset and distress and find out what treatment options may be useful.

Why have I been invited?

As a person with chronic musculoskeletal pain, we would be interested in hearing your views and experiences. We would like to talk to people who have found that their pain has impacted on their mood. Sometimes doctors talk about this as 'depression', others may talk about low mood, or stress. We would like to talk to you about how you think about these feelings, and what you do to manage them.

Do I have to take part?

You do not have to take part in this study. Participation is entirely voluntary. You are also free to withdraw at any time during the interview, without giving any reason and without your rights, treatment or the care you receive being affected. You may withdraw your interview data up to four weeks after the date consent was provided (i.e. the date of your interview); this data will then be destroyed.

What does taking part involve?

You are invited to participate in an interview conducted by the study researcher. The interview will be held at a place and time convenient to you at your home address, Keele University or another place convenient to you; the interview could take place by telephone or a video platform (such as 'Microsoft Teams' or 'Google Hangouts') if you wish.

You will be asked to sign a consent form stating that you agree to take part in the study. This is to record that you have read this information sheet, understand what the research study is about, and that you agree to take part as described. Before starting the interview, you will be asked to complete a short questionnaire (the Brief Pain Inventory) that asks about your pain experience.

During the interview we will discuss your personal experiences of emotional distress, with a particular focus on its relationship to pain, and if you sought help. With your permission, we will audio-record the interview and write this up for our analysis. We expect the interview to last around 45 minutes.

Will I be reimbursed for my time?

You will be offered a £20 voucher to recompense your time, and reasonable travel expenses will be reimbursed.

What are the benefits/risks of taking part?

Whilst there are no immediate benefits of taking part, some individuals may find it helpful to talk through their experiences. Your contribution will be valuable in enabling us to learn more about emotional distress in people with pain. Your data will help us to conduct future research with the aim to improve healthcare services in supporting people with pain and distress.

Whilst there are no expected risks, some individuals may find talking about their personal experiences distressing. If during the interview you feel distressed or get upset, you can ask to take a break or end the interview. The researcher will provide information about services that you may wish to access (e.g. counselling services).

How will we use information about you?

Your participation will be kept confidential so no one else will know you have taken part. Royal Holloway, University of London, is the sponsor for this study. We will need to use information from you for this research project. This information will include your age, gender, type of pain condition and the data from the interview. People will use this information to do the research. People who do not need to know who you are will not be able to see your name or contact details. Your data will have a code number instead. We will keep all information about you safe and secure. Once we have finished the study, we will keep some of the data so we can check the results. We will write our reports in a way that no-one can work out that you took part in the study.

Prior to the interview, the researcher will ask you to sign a consent form to give your written permission to audio-record the interview and type it up. The anonymised research data will be stored for a minimum of 10 years. When writing up the study results, we may use direct quotes from you. These quotes will be completely anonymised by using a study ID code instead of your name and removing identifiable personal information (for example, the name of your place of work). You will not be able to be identified from these quotes.

Please note, in exceptional circumstances during the course of your participation in this research, where a research team member has any safety concerns about you or others the team may need to breach confidentiality to share these concerns with appropriate services.

Where can I find out more about how my information is used?

You can find out more about how we use your information

- At https://www.hra.nhs.uk/information-about-patients/
- Our leaflet available from http://www.hra.nhs.uk/patientdataandresearch
- By asking one of the research team
- By sending an email to Professor Carolyn Chew-Graham at c.a.chew-graham@keele.ac.uk
- By ringing Professor Carolyn Chew-Graham on 01782 734 717

What will happen to the results of the study?

We will write up what we find out from the interviews for a professional research journal so that the findings are shared with other academics and healthcare professionals. We also intend to present the findings at conferences that are open to members of the public and circulate summary reports to local voluntary organisations. Your personal information will not be used in any of these documents or presentations. This study will form an important foundation to develop further research into managing emotional distress for people with chronic musculoskeletal conditions.

Who is funding the research?

This research project is funded by Versus Arthritis (grant number 22454).

Has the research study received ethical approval?

Yes, this study has been given favourable ethical opinion by the South East Scotland Research Ethics Committee.

Who do I contact if there is a problem?

To speak to the lead qualitative researcher and Principal Investigator, please contact:

Professor Carolyn Chew-Graham
School of Medicine, Keele University, ST5 5BG
Email: c.a.chew-graham@keele.ac.uk
Telephone: 01782 734 717

To speak to the Chief Investigator of the De-STRESS study please contact:

Professor Tamar Pincus
School of Psychology, Royal Holloway University of London, SW
Email: t.pincus@rhul.ac.uk
Telephone: 01784 443 523

If you have any questions or concerns about taking part in this research, you can also contact Royal Holloway, University of London's Director of Research and Innovation on: researchservices@rhul.ac.uk.

As this research has been conducted through NHS services, you can contact your local Patient Advice and Liaison Service (PALS). Visit https://www.nhs.uk/nhs-services/hospitals/what-is-pals-patient-advice-and-liaison-service/ to find your local service.

Alternatively, you can contact NHS England on Tel: 0300 311 2233, email: england.contactus@nhs.net.

For all other enquiries, or to arrange an interview, please contact the study researcher:

Dr Noureen Shivji
School of Medicine, Keele University
Email: n.shivji@keele.ac.uk

Qualitative topic guide for people with pain

Developing a patient-centred evidence-based intervention to address distress in the context of chronic MSK pain.

<u>Topic Guide for People with pain</u>

- Introduction and consent
- Demographics: age, gender, level of education reached, (previous) employment, rural/urban/inner city, GP details
- Main prompts

Can you tell me about the pain(s) you have? How does this affect you? How is your mood affected by your pain?

Sometimes we use the term 'emotional distress' to describe how people feel when they also have pain…what do you understand by 'distress'?

What do you do if you feel upset/distressed?

Do you seek help differently for emotional and physical problems?

Do you seek help when you feel emotionally distressed? From whom (explore family, friends, health/social care; other).

Have you talked to any healthcare professionals (HCPs) about how you feel – your mood, being upset?

What made you go to see the (HCP)? [or what would make you consult a HCP?]

Topic guide v1.0 28may2020

Sometime when people have pain, and are upset, a doctor might suggest a diagnosis of 'depression' – have you any experience of this? How did you feel about this diagnosis? How helpful is the label of 'depression'?

What sort of treatment(s) have you been offered? (explore where delivered, who by…) What helped/didn't help and why?

What help would you like to receive for both your pain and distress/low mood?

We are designing a new treatment to help people manage their mood – what do you think should be included in this?
How would you feel if some of this new treatment was delivered online/via an app.? Would you want someone (a HCP/ other) to help you with such an app?

Anything else you would like to add?

Close interview: Thanks, re-check consent, arrange reimbursement.

Qualitative GP recruitment email

Recruitment email for GPs

Dear Dr XX

Developing a patient-centred evidence-based intervention to address distress in the context of chronic musculoskeletal pain.

We would like to invite you to participate in the above study. I enclose an information sheet which outlines the aims of the study and what participation will involve.

In summary, the study involves:

i) Practices supporting recruitment of adults who musculoskeletal pain, inviting them to participate in an interview with a member of the research team

ii) Interviewing GP partners or salaried GPs who see people with chronic MSK pain, so that we can better understand their experiences managing people with emotional distress and pain. The interview will last approximately half an hour in your place of preference, of over the telephone, or using other software (such as 'Microsoft Teams'.
Financial renumeration is provided for your time at a rate of £88 per hour.

Further details are attached in the participant information sheet. I would be very grateful if you would be willing to take part in this study. If you are interested, please email the study researcher at XXXXX. If you do so, you will have the chance to find out more about the study before coming to any decision. You would be under no obligation to take part.

The use of email to recruit participants for this study has been approved by the Keele University Research Ethics Committee as well as the NHS Health Research Authority.

Best Regards,
Professor Carolyn Chew-Graham
Professor of General Practice Research

Researcher details XXX

School of Primary, Community and Social Care
Keele University

Qualitative GP information leaflet

De-STRESS
pain

Participant Information Sheet for General Practitioners

We invite you to take part in our research study entitled "***Developing a patient-centred evidence-based intervention to address distress in the context of chronic musculoskeletal pain.***"

Before you decide whether or not you wish to take part, it is important for you to understand why this research is being done and what it will involve.

Please take your time to read this information sheet carefully.
Should you have any questions please contact Professor Chew-Graham
(Email: c.a.chew-graham@keele.ac.uk)

Summary:

- This study is being carried out by researchers at Keele University, Royal Holloway (University of London) and University of Southampton
- Your participation is voluntary
- The research has been approved by Keele University Research Ethics Committee and has HRA approval
- Participation involves a single interview, lasting approximately 30 minutes, at a location convenient for you
- Your consent to take part will be recorded prior to the interview
- Your personal information will be made anonymous so that what you say in the interview cannot be linked back to you

Who is carrying out this research?

This study is being carried out by Professor Carolyn Chew-Graham at the School of Primary, Community and Social Care, Keele University, along with researchers from the Universities of Southampton and London. The study is funded by Versus Arthritis (grant number 22454).

What is the purpose for this research?

The reason for the research is to find out how people with pain due to musculoskeletal conditions talk about their mood and distress, and how general practitioners (GPs) what they think pain-related distress is, and what sorts of treatments they offer people. When we understand what patients and GPs think about pain-related distress, we will design a specific treatment to help.

Why have I been invited?

As a GP partner or salaried GP who works with patients with chronic pain, we would be interested in hearing your views and experiences.

Do I have to take part?

You do not have to take part in this study. Participation is entirely voluntary. You are also free to withdraw at any time during the interview, without giving any reason.

You may withdraw interview data up to four weeks after the date consent was provided (i.e. the date of your interview); this data will then be destroyed.

What does taking part involve?

You will be invited to attend an interview conducted by the study research (name). The interview will be held at a place and time convenient to you (at your work address or Keele University); alternatively, the interview could be conducted over the telephone or a platform such as 'Microsoft Teams'.

With your permission, we will audio-record the interview and write this up for our analysis. We expect the interview to last approximately half an hour.

You will be asked to sign a consent form stating that you agree to take part in the study. This is to record that you have read this information sheet, understand what the research study is about, and that you agree to take part as described. If the interview is conducted remotely, we will ask you to send your signed consent form to the research team at Keele University. We will record your consent on the recorded interview.

During the interview we will discuss your experiences managing emotional distress in people with chronic musculoskeletal disorders, with a particular focus on how they present and barriers and facilitators to care. We will use this information to develop an intervention specifically for people who have distress as well as chronic musculoskeletal pain.

Will I be reimbursed for my time?

You will receive £88 per hour to recompense your time and reasonable travel expenses will be reimbursed.

What are the benefits/risks of taking part?

There are no immediate benefits of taking part. Your contribution will be valuable in enabling us to learn more about managing emotional distress in people with pain and distress.

Whilst there are no expected risks, some individuals may find talking about experiences with patients distressing.

Who will have access to information about me?

Your participation will be kept confidential (in exceptional circumstances confidentiality may need to be breached). Keele University is the sponsor for this study. As we will be collecting information from you as part of the study, Keele University will act as the data controller for this information. This means that the research team (on behalf of Keele University) are responsible for looking after your information and using it properly. Keele University will keep identifiable information about you for up to 6 months.

Your rights to access, change or move your information are limited, as we need to manage your information in specific ways in order for the research to be reliable and accurate. If you withdraw from the study, we will keep the information about you that we have already obtained. To safeguard your rights, we will use the minimum personally-identifiable information possible. Your personal data will be processed in accordance with Keele University Standard Operating Procedures and in line with the General Data Protection Regulation (GDPR). You can find out more about how we use your information at https://www.keele.ac.uk/informationgovernance/checkyourinformationisbeinghandledcorrectly/privacynotice-researchparticipants/.

Prior to the interview, the researcher will request your written permission to audio-record the interview and then transcribe it. The anonymised research data will be stored for a minimum of 10 years.

Please note, in exceptional circumstances during the course of your participation in this research, where a research team member has any safety concerns about you or others the team may need to breach confidentiality to share these concerns with appropriate services.

What will happen to the results of the study?

We will write up what we find out from the interviews for a professional research journal so that the findings are shared with other academics and healthcare professionals. We also intend to present the findings at conferences that are open to members of the public and circulate summary reports to local voluntary organisations. Your personal information will not be used in any of these documents or presentations. This study will form an important foundation to develop further research into managing emotional distress in people with musculoskeletal pain.

Who is funding the research?

This research project is funded by Versus Arthritis (grant number 22454).

Has the research study been ethically approved?

Yes, this study has been ethically approved by Keele University's Keele University's Research Ethics Committee (REC) and the NHS Health Research Authority (HRA).

VERSUS ARTHRITIS   University of Southampton   Royal Holloway University of London   Keele University

Who do I contact if there is a problem?

To speak to the lead study supervisor, please contact:

Professor Carolyn Chew-Graham
School of Primary, Community and Social Care, Keele University, ST5 5BG
Email: c.a.chew-graham@keele.ac.uk
Telephone: 01782 734 717

If you have any questions or concerns about taking part in research you can also contact Keele University's Head of Project Assurance: research.governance@keele.ac.uk. Alternatively, you can contact NHS England on Tel: 0300 311 2233, email: england.contactus@nhs.net.

For all other enquiries or to arrange an interview please contact the researcher XXXX

School of Primary, Community and Social Care, Keele University, ST5 5BG
Email: XXXX

Qualitative GP topic guide

Developing a patient-centred evidence-based intervention to address distress in the context of chronic MSK pain.

Topic Guide for GPs

- Introduction and consent

- Demographics: age, area worked, number of sessions worked clinically, years of experience as a GP, role (partner/salaried and extra roles eg GPwSI/CCG role etc), areas of interest/expertise

- Main prompts:

What sort of management do you offer a person with pain who is 'distressed'?
How does that compare to when you think the patient is 'depressed'?
Can you give an example of a recent patient you saw and how you dealt with this?

How do you think pain and distress are linked?
Is this the same for all types of pain, whatever the cause?
Is there anything particular that you take into consideration when someone with MSK pain presents? Can you give an example of a patient you saw recently, and you handled this?

How to people with pain and distress present?
Do people seek help differently for emotional and physical problems?
What about when problems are combined?
What are the facilitators and barriers to you in identifying and managing emotional distress in people with MSK pain?

Is this the same for other GPs? What about other clinicians (pain specialists; physiotherapists)?

How do you distinguish between 'distress' and 'depression'?
When might you apply the term 'depression' to a person with MSK pain?

What sort of help do people with pain and distress come to you for?

What sorts of referral pathways/ management options are available to you for your patients with pain and depression?
How useful are the services available?
How acceptable are the services that are available to patients?
Anything that you would like to see, that currently isn't available?

How might an intervention be delivered 'on-line'/app. to patients? Is this something you might facilitate?

Anything else you would like to add?

Close interview: Thanks, re-check consent, arrange reimbursement.

Qualitative table of themes

Main theme	Subtheme	Exemplar quote (s)
The intrinsic link between pain and distress	Pain directly impacts mood	Okay, I would say that it is distressing, my pain definitely, because now I feel like it's a constant (P7)
	No pain equals no distress	If my treatment works and I have been through a lot of treatments in this, if it works I feel that I pretty much have a perfect life (P2)
	Distress changes in relation to pain experience	No, in my personal opinion I don't actually think that the person should label as depressed, because this is a symptom that maybe comes and goes with pain increases or decreases, it depends sometimes on the level of the pain (P7)
	Distress and depression can co-exist	It may be that their anxiety and depression is there, but not such a big issue if their pain is controlled. Hard to distinguish between purely just the distress and purely mental health issues. I think it's sometimes, often is more of a blurred situation and there may be elements of both (GP4)
	Distress can become depression over time	I think depression comes out of distress. I don't think it works the other way. I think distress is the physical feeling of pain which becomes so constant that it can only really manifest itself in depression (P21)
	A person's context is important to understanding low mood	I have asked people if the pain was better would they feel better sort of thing and some people, then yes. But other people it's a no because of X, Y and Z that's also going on or how they otherwise feel. And with them the joint pain is sort of adding to it, but not the sole cause [of low mood] (GP3)
Components of pain-related distress	The impact of pain on physical function, and subsequently identity	I need so yeah it's affected my well-being in that respect but also physically as well, not being able to do what I want to do. I used to be a runner and I can't run any more and that really has devastated me (P3)<br><br>Yes I think it increases low mood. Because it's quite dominant, it's a sort of constant feature and it stops things, it stops me being able to do things. I mentioned that the DIY in the kitchen, and also it stops me kind of doing stuff in the community that I like (P11).

	Positivity outside of pain	Not really depression, I'm not a depressive sort of person, I'm generally quite optimistic and cheerful. But now and again it makes me fed up and cross but I wouldn't say depressed (P1)<br><br>If you'd asked the other lady about her life, the one that I weaned her off the medication, she was actually quite, you know, solid with her relationships, she was working and you know it wasn't taking over but she was stressed about this thing but she could happily talk about other bits of her life in a positive way (GP5)
	Acceptance	Going back to my medical history, I've had severe depression and I've learnt to try and look on the positive side and deal with things that I can't do. There are things I know I can't do and so I just have to realise I can't do them. I don't tend to get frustrated now (P14)
	Coping	I think I've become mentally stronger. I just try and do what I can, like I've said before, within my own limitations. Everybody changes, don't they? It's a hard question to think about how I've changed but I've just coped with it as best I can. I've developed coping strategies (P14)
Identifying types of low mood in a consultation for pain	Difficult to distinguish between pain-related distress and depression	I like your distinction between distress and depression because I think it's often very difficult to differentiate... people with chronic pain they become... their mood is lower and they become anxious so it's kind of difficult to differentiate it (GP12)
	Screening tools provide an objective assessment	You could use PHQ 9 or other scores to help and it's that assessment, objective and subjective assessment that you think somebody is potentially being depressed which is obviously severe (GP4)<br><br>If I'm suspecting depression I probably would set onto the PHQ 9 template and do a proper mental health questionnaire and try and gauge that better (GP12)
	Issues with the PHQ-9 and pain	Yes, I mean you can use things like PHQ9 but those are screening type questionnaires and I'm probably not one to use them because I find that everyone scores very highly, and it equates to depression (GP8)
	Clinical conversations facilitate understanding	I would say the first and the most important thing is for them to have a good therapeutic relationship with a GP or with a clinician of some sort. I would say that's number one; to have a clinician that they will sit down and engage in a sensible conversation with. The clinician listens to them, listens to their pain and distress, understands how it's affecting their life, shows empathy and sympathy and also for then the patient to listen to the doctor and listen to all the different options that that doctor is putting on the table. I think having

		excellent communication and relationship building skills, gaining a relationship of trust between the GP and the patient, is absolutely essential (GP19)
	Continuity and knowing the patient	Continuity is an important thing in this and we haven't mentioned, so for me I've known patients for 20 years so it makes it much, much easier for me, hopefully for the patient, but certainly for me. Because I understand them, I know where they're coming from, I know their family, I know the context, I know what's happened to them you know. And that makes it so much easier than for someone who's seen them from new (GP15)

Quantitative cover letter

Dear <<Title>> <<First name>> <<Surname>> <<Date>>

At <<GPpractice>>, we are participating in a study funded by Versus Arthritis titled ***Developing a patient-centred evidence-based intervention to address distress in the context of chronic musculoskeletal pain.*** To undertake this study, <<GPpractice>> are working with researchers from the University of Southampton and Keele University.

We have identified that you meet the criteria for this study. The study is recruiting people with chronic pain, people with depression, and people with a mix of both conditions.

This study has been reviewed by South Central – Hampshire B Research Ethics Committee and given favourable ethical opinion.

Taking part in the study involves completing a research questionnaire. This questionnaire can be completed on paper (included in the study pack with a freepost return envelope) or online at https://tinyurl.com/destresspain. The questionnaire asks you about your experiences of pain (if relevant) and mood. Enclosed is an information leaflet which explains the study in more detail.

Your participation is voluntary, and it is up to you whether you take part. If you have any questions about this research, please contact the study team at destress@soton.ac.uk.

Please be assured that unless you decide to complete and return the questionnaire, no personal information (e.g., your name, address, and diagnoses) will be shared with the researchers.

Thank you for considering participating in this study. If you do not wish to take part, this will not affect the care you receive from the practice in any way.

Yours sincerely

<<GPpractice>>

Quantitative patient information leaflet

VERSUS ARTHRITIS

UNIVERSITY OF Southampton

Keele UNIVERSITY

Information leaflet

De-STRESS *pain*

Exploring how peoples' experiences of pain relate to emotions and experiences of stress: A questionnaire study

We invite you to take part in our research study. Before you decide whether or not you wish to take part, it is important to understand why this research is being done and what it will involve.

Please take your time to read this information sheet carefully.

Should you have any questions please contact the study team (destress@soton.ac.uk) or the principal investigator Dr Adam Geraghty (a.w.geraghty@soton.ac.uk)

Summary

This study is being carried out by researchers at the University of Southampton and Keele University.

Taking part is voluntary.

The study has been reviewed by South Central – Hampshire B Research Ethics Committee who have given a favourable opinion of the study.

Participation involves completing a single questionnaire asking about your recent experiences of mood and, if relevant, chronic pain.

The questionnaire should be completed online or returned by post in the provided freepost envelope.

Your personal information will be made anonymous.

Who is carrying out this research?

This study is being carried out a team of researchers from the University of Southampton and Keele University. The study is funded by Versus Arthritis (grant number 22454).

Why are we doing this research?

Pain will often affect our mood and how we feel. When people have experienced pain for a long time, they may also experience stress and low mood. This may be labelled depression. Our team are exploring whether it is useful to think about feelings and concerns associated with pain differently, using different labels. That is why we are asking people with and without pain and with and without depression to complete our questionnaire.

Why have I been invited?

You have been invited because your GP records indicate that you experience either chronic musculoskeletal pain, depression, or both.

What does taking part involve?

You are invited to complete a questionnaire. The questionnaire will cover topics of pain (if relevant to you), low mood, depression, anxiety, frustration, anger, life events and optimism.

Do I have to take part?
You do not have to take part in this study. Participation is entirely voluntary. You are also free to withdraw at any time whilst completing the questionnaire, without giving any reason and without your rights, treatment, or the care you receive being affected. You may withdraw your questionnaire data up to four weeks after the date consent was provided (i.e. the date of completing the questionnaire); this data will then be destroyed.

How do I take part?
You can take part by simply completing the enclosed questionnaire and returning it in the freepost envelope provided. The questionnaire will take about 30 minutes to complete.

Consent
Whether you complete this questionnaire online or on paper, you will be asked whether you consent to take part. Giving consent means that you agree that:

- You have read this information leaflet.
- You acknowledge that your participation is voluntary, and that you have the right to withdraw data up to 4 weeks after returning the questionnaire.
- You are happy to take part in this study.

⇒ If you are completing the questionnaire on paper, please make sure you complete and return the enclosed consent form. Without this, we cannot use the answers you give us.

There is an additional consent section at the end of the questionnaire relating to future research. In this section you will be asked if you consent to being contacted for future research on this topic. Please note that this question is optional. If you do not wish to be contacted for future research, leave this box blank. Then you can complete this questionnaire only, and will not be contacted further.

What are the benefits/risks of taking part?
Whilst there are no immediate benefits of taking part, your contribution will be valuable in helping us to learn more about emotional distress in people with pain, and how this differs from depression. Your data will help us to conduct future research with the aim to improve healthcare services in supporting people with pain and distress.

Whilst there are no expected risks, some individuals may find answering questions about personal experiences distressing. If during the questionnaire you feel distressed or get upset, you can take a break and finish the questionnaire later or decide to not answer any more questions. You can also contact the Samaritans or visit the MIND website using the details given at the end of this document.

How will we use information about you?
Your participation will be kept confidential so no one else will know you have taken part. The University of Southampton is the sponsor for this study. We will need to use information from you for this research project. This information will include your age, gender, type of pain condition and the data from the questionnaire. People will use this information to do the research. People who do not need to know who you are will not be able to see your name or contact details. Your data

will have a code number instead. We will keep any identifiable information about you in a separate database to your answers to the questionnaire. We will only access the identifiable information if we have concerns for your safety and need to contact your GP. We will keep all information about you safe and secure. Paper copies of the questionnaire will be stored in locked filing cabinets at the University of Southampton, accessed only by people with permission. Electronic data will be stored on password protected, private, secure severs at the University of Southampton. Once we have finished the study, we will keep some of the data so we can check the results. We will write our reports in a way that no-one can work out that you took part in the study.

The anonymised research data will be stored for a minimum of 10 years. Your data will be given a study ID code instead of your name and identifiable personal information will be removed. You will not be able to be identified from your data. This anonymous research data may be available to approved researchers at the University of Southampton or Keele University for further analysis.

Please note that there are a few questions that ask about suicidal ideation and harm to yourself in the questionnaire. If you answer these specific questions in such a way that the research team has safety concerns, then the team may need to breach confidentiality to share these concerns with your GP.

Our privacy notice for research participants provides more information on how the University of Southampton collects and uses your personal data when you take part in one of our research projects and can be found at http://www.southampton.ac.uk/assets/sharepoint/intranet/ls/Public/Research%20and%20Integrity%20Privacy%20Notice/Privacy%20Notice%20for%20Research%20Participants.pdf

If you have any questions about how your personal data is used, or wish to exercise any of your rights, please consult the University's data protection webpage (https://www.southampton.ac.uk/legalservices/what-we-do/data-protection-and-foi.page) where you can make a request using our online form. If you need further assistance, please contact the University's Data Protection Officer (data.protection@soton.ac.uk).

Where can you find out more about how your information is used?
You can find out more about how we use your information:

- At https://www.hra.nhs.uk/information-about-patients/
- The leaflet available from http://www.hra.nhs.uk/patientdataandresearch
- By sending an email to Dr Adam Geraghty at a.w.geraghty@soton.ac.uk

What will happen to the results of the study?
We will write up what we find out from the questionnaires for a professional research journal so that the findings are shared with other academics and healthcare professionals. We also intend to present the findings at conferences that are open to members of the public and circulate summary reports to local voluntary organisations. Your personal information will not be used in any of these documents or presentations. This study will form an important foundation to develop further research into managing emotional distress for people with chronic musculoskeletal conditions. The results will be available on the Versus Arthritis website after study completion, expected end of 2024. The direct link for the study is: https://www.versusarthritis.org/research/our-current-research/our-current-research-

projects/chronic-pain-improving-detection-and-treatment-of-distress-in-people-with-chronic-msk-pain/

Who is funding the research?

This research project is funded by Versus Arthritis (grant number 22454).

Has the research study received ethical approval?

Yes, this study has been reviewed by the South Central – Hampshire B Research Ethics Committee who have given a favourable opinion of this study.

Who do I contact if there is a problem?

To speak to the lead quantitative researcher and Principal Investigator, please contact:

Dr Adam Geraghty, Associate Professor
Faculty of Medicine, University of Southampton
Email: a.w.geraghty@southampton.ac.uk
Telephone: 023 8059 1770 (please note that this is an answering machine and messages will be picked up weekly).

To speak to the Chief Investigator of the De-STRESS pain study please contact:

Professor Tamar Pincus
Dean of the Faculty of Environmental and Life Sciences, University of Southampton
Email: t.pincus@soton.ac.uk

Who can I contact outside of the research team?

If you have any questions or concerns about taking part in this research, you can also contact University of Southampton's Research Governance Manager by emailing rgo@soton.ac.uk or calling 023 8059 5058. As this research has been conducted through NHS services, you can contact your local Patient Advice and Liaison Service (PALS). Visit https://www.nhs.uk/nhs-services/hospitals/what-is-pals-patient-advice-and-liaison-service/ to find your local service. You can also contact NHS England on Tel: 0300 311 2233, email: england.contactus@nhs.net.

Support

If you have found anything in this questionnaire distressing and would like someone to talk to, the Samaritans can be contacted at any time by calling 116 123 for free, or by emailing jo@samaritans.org. Information and support about mental health can also be found on MIND's website: www.mind.org.uk.

You can complete the questionnaire either online or on paper.

Online:

⇒ Go to: https://tinyurl.com/destresspain
⇒ You will need to enter your participant ID number (found on the enclosed consent form).

Paper:

⇒ Please complete the consent form and questionnaire, and return both in the freepost envelope provided.

If you have any queries, please contact the study team on destress@soton.ac.uk

Quantitative cross-sectional survey

De-STRESS
pain

Questionnaire

VERSUS ARTHRITIS
University of Southampton
Keele University

IRAS number: 303304 1 De-STRESS questionnaire v1.8 13th June 2022

INSTRUCTIONS FOR THIS QUESTIONNAIRE

Please complete this form in pen and block capitals.

The aim of this questionnaire is to find out more about you, your mood, and emotions, and if you experience it, chronic pain. Importantly, this questionnaire has been sent to people who both do and do not experience chronic pain. Some questions may not seem as relevant to you as others, but please answer all the questions unless the instructions ask you to do something else. There are no right or wrong answers. All your answers will be kept confidential.

The questions ask you to either check a box, circle a number, or write your response. If you have any questions, or need help completing this questionnaire, please contact the De-STRESS pain study team at the University of Southampton at destress@soton.ac.uk.

For more information, please see the enclosed information leaflet.

Thank you for your help with this research study. Please continue and fill in this questionnaire.

Section A – About you

Please write in capitals below

1. Your full name:

____

2. The best telephone number to contact you on:

____

3. Your email address:

____

4. The name of your GP surgery:

____

5. What is your date of birth? *(please answer dd/mm/yyyy e.g., 26th May 1963 would be 26/05/1963)*

☐☐ / ☐☐ / ☐☐☐☐

6. Are you:

☐	☐	☐	☐
Male	Female	Non-binary	Prefer not to say

7. What is your current employment status?

☐	☐	☐	☐	☐
Full-time paid employment	Part-time paid employment	Volunteer work	Retired	Unemployed

8. What is your current, or most recent, job title (even if you are not currently working)?

____

**9. What is the highest educational qualification you have?**			
High school (e.g., GCSE, BTEC)	☐	University post-graduate (e.g., MSc, MA, PGCE)	☐
College (e.g., A-Level or equivalent)	☐	Doctorate (e.g., PhD, MD)	☐
University undergraduate (e.g., BSc, BA)	☐		
Other: ______________________________			

10. What is your ethnic group? *Choose one option that best describes your ethnic group or background.*

**White**		**Mixed/Multiple ethnic groups:**	
Welsh/English/Scottish Northern Irish/British	☐	White and Black Caribbean	☐
Irish	☐	White and Black African	☐
Gypsy or Irish Traveller	☐	White and Asian	☐
Any other White background, please describe:		Any other Mixed/Multiple ethnic background, please describe:	
______________________		______________________	
**Asian/Asian British**		**Black/African/Caribbean/Black British**	
Indian	☐	African	☐
Pakistani	☐	Caribbean	☐
Bangladeshi	☐	Any other Black/African/Caribbean background, please describe:	
Chinese	☐	______________________	
Any other Asian background, please describe:		**Any other ethnic group, please describe:**	
______________________			
**Other ethnic group**			
Arab	☐	______________________	

Section B – About your health

These questions ask about different health conditions. Please answer every question. If you do not experience the condition, please check the corresponding box.

Musculoskeletal pain

1. Do you experience musculoskeletal pain (pain in your muscles, joints or bones, or pain from a condition such as fibromyalgia, osteoarthritis, or rheumatoid arthritis)?

☐ Yes ☐ No

1a. What types of musculoskeletal pain do you have?

____

____

____

☐ I do not have musculoskeletal pain

1b. How long is it since you last had a whole month without any pain?

☐	☐	☐	☐
Less than 3 months	3 to 6 months	7 to 12 months	1 to 2 years
☐	☐	☐	☐
3 to 5 years	6 to 10 years	10+ years	I do not have musculoskeletal pain

Low mood and depression				
**2.** Are you currently experiencing low mood or depression?				
☐ Yes	☐ No			
**2a.** Have you ever been diagnosed with depression?				
☐ Yes	☐ No			
**2b.** How many years have you experienced episodes of low mood or depression?				
☐ Less than 1 year	☐ 1 year	☐ 2 years	☐ 3 years	
☐ 4 years	☐ 5 years	☐ More than 5 years	☐ I have never experienced low mood or depression	
**2c.** If you have experienced low mood or depression *and* you have musculoskeletal pain, was this before the onset of your pain?				
☐ Yes	☐ No	☐ Not sure	☐ I don't have musculoskeletal pain	☐ I have never experienced depression

Anxiety				
**3.** Are you currently experiencing anxiety?				
	☐ Yes	☐ No		
**3a.** Have you ever been diagnosed with anxiety?				
	☐ Yes	☐ No		
**3b.** How many years have you experienced episodes of anxiety?				
☐ Less than 1 year	☐ 1 year	☐ 2 years	☐ 3 years	
☐ 4 years	☐ 5 years	☐ More than 5 years	☐ I have never experienced anxiety	
**3c.** If you have experienced anxiety *and* you have musculoskeletal pain, was this before the onset of your pain?				
☐ Yes	☐ No	☐ Not sure	☐ I don't have musculoskeletal pain	☐ I have never experienced anxiety

Antidepressants				
**4.** Are you currently prescribed antidepressants?				
☐ Yes	☐ No			
**4a.** Are your antidepressants prescribed to manage:				
☐ Mood	☐ Pain	☐ Both mood and pain	☐ Not sure	☐ I am not prescribed antidepressants
**4b.** Do you feel that the antidepressants are helping to manage your condition?				
☐ Yes, a lot	☐ Yes, moderately	☐ Yes, a little	☐ No	☐ I am not prescribed antidepressants

Section C – Questions about mood

1. **Over the last 2 weeks how often have you been bothered by any of the following problems?** *(Use "✓" to indicate your answer)*	**Not at all**	**Several days**	**More than half the days**	**Nearly every day**
a) Little interest or pleasure in doing things	☐	☐	☐	☐
b) Feeling down, depressed, or hopeless	☐	☐	☐	☐
c) Trouble falling or staying asleep, or sleeping too much	☐	☐	☐	☐
d) Feeling tired or having little energy	☐	☐	☐	☐
e) Poor appetite or overeating	☐	☐	☐	☐
f) Feeling bad about yourself – or that you are a failure or have let yourself or your family down	☐	☐	☐	☐
g) Trouble concentrating on things, such as reading the newspaper of watching television	☐	☐	☐	☐
h) Moving or speaking so slowly that other people could have noticed. Or the opposite – being so fidgety or restless that you have been moving around a lot more than usual	☐	☐	☐	☐
i) Thoughts that you would be better off dead or of hurting yourself in some way	☐	☐	☐	☐

If you ticked any problems, how difficult have these problems made it for you to do your work, take care of things at home, or get along with other people?

**Not difficult at all**	**Somewhat difficult**	**Very difficult**	**Extremely difficult**
☐	☐	☐	☐

2. **The following is a list of questions about various complaints and symptoms you may have. Each question refers to the complaints and symptoms that you had in the past week (the past 7 days, including today). Complaints you had before then, but no longer had during the past week, do not count.**

Please indicate for each complaint how often you noticed that you had it in the past week by putting an "X" in the box under the answer that is most appropriate.

**During <u>the past week</u>, did you suffer from:**	**No**	**Some-times**	**Regularly**	**Often**	**Very often or constantly**
a) dizziness or feeling light-headed?	☐	☐	☐	☐	☐
b) painful muscles?	☐	☐	☐	☐	☐
c) fainting?	☐	☐	☐	☐	☐
d) neck pain?	☐	☐	☐	☐	☐
e) back pain?	☐	☐	☐	☐	☐
f) excessive sweating?	☐	☐	☐	☐	☐
g) palpitations?	☐	☐	☐	☐	☐
h) headache?	☐	☐	☐	☐	☐
i) a bloated feeling in the abdomen?	☐	☐	☐	☐	☐
j) blurred vision or spots in front of your eyes?	☐	☐	☐	☐	☐
k) shortness of breath?	☐	☐	☐	☐	☐
l) nausea or an upset stomach?	☐	☐	☐	☐	☐

Continued on next page

Question 2 continued.
Please indicate for each complaint how often you noticed that you had it in the past week by putting an "X" in the box under the answer that is most appropriate.

**During the past week, did you suffer from:**	**No**	**Some-times**	**Regularly**	**Often**	**Very often or constantly**
m) pain in the abdomen or stomach area?	☐	☐	☐	☐	☐
n) tingling in the fingers?	☐	☐	☐	☐	☐
o) pressure or a tight feeling in the chest?	☐	☐	☐	☐	☐
p) pain in the chest?	☐	☐	☐	☐	☐
q) feeling down or depressed?	☐	☐	☐	☐	☐
r) sudden fright for no reason?	☐	☐	☐	☐	☐
s) worry?	☐	☐	☐	☐	☐
t) disturbed sleep?	☐	☐	☐	☐	☐
u) a vague feeling of fear?	☐	☐	☐	☐	☐
v) lack of energy?	☐	☐	☐	☐	☐
w) trembling when with other people?	☐	☐	☐	☐	☐
x) anxiety or panic attacks?	☐	☐	☐	☐	☐

Continued on next page

Question 2 continued.
Please indicate for each complaint how often you noticed that you had it in the past week by putting an "X" in the box under the answer that is most appropriate.

**During the past week, did you feel:**	**No**	**Some-times**	**Regularly**	**Often**	**Very often or constantly**
y) tense?	☐	☐	☐	☐	☐
z) easily irritated?	☐	☐	☐	☐	☐
aa) frightened?	☐	☐	☐	☐	☐
bb) that everything is meaningless?	☐	☐	☐	☐	☐
cc) that you just can't do anything anymore?	☐	☐	☐	☐	☐
dd) that life is not worthwhile?	☐	☐	☐	☐	☐
ee) that you can no longer take any interest in the people and things around you?	☐	☐	☐	☐	☐
ff) that you can't cope anymore?	☐	☐	☐	☐	☐
gg) that you would be better off if you were dead?	☐	☐	☐	☐	☐
hh) that you can't enjoy anything anymore?	☐	☐	☐	☐	☐
ii) that there is no escape from your situation?	☐	☐	☐	☐	☐
jj) that you can't face it anymore?	☐	☐	☐	☐	☐

Continued on next page

Question 2 continued.
Please indicate for each complaint how often you noticed that you had it in the past week by putting an "X" in the box under the answer that is most appropriate.

**During the past week did you:**	**No**	**Some-times**	**Regularly**	**Often**	**Very often or constantly**
kk) no longer feel like doing anything?	☐	☐	☐	☐	☐
ll) have difficulty in thinking clearly?	☐	☐	☐	☐	☐
mm) have difficulty in getting to sleep?	☐	☐	☐	☐	☐
nn) have any fear of going out of the house alone?	☐	☐	☐	☐	☐
**During the past week:**					
oo) did you easily become emotional?	☐	☐	☐	☐	☐
pp) were you afraid of anything when there was really no need for you to be afraid? *(for instance animals, heights, small rooms)*	☐	☐	☐	☐	☐
qq) were you afraid to travel on buses, streetcars/trams, subways or trains?	☐	☐	☐	☐	☐
rr) were you afraid of becoming embarrassed when with other people?	☐	☐	☐	☐	☐
ss) did you ever feel as if you were being threatened by unknown danger?	☐	☐	☐	☐	☐
tt) did you ever think "I wish I was dead"?	☐	☐	☐	☐	☐
uu) did you ever have fleeting images of any upsetting event(s) that you have experienced?	☐	☐	☐	☐	☐
vv) did you ever have to do your best to put aside thoughts about any upsetting events(s)?	☐	☐	☐	☐	☐
ww) did you have to avoid certain places because they frightened you?	☐	☐	☐	☐	☐
xx) did you have to repeat some actions a number of times before you could do something else?	☐	☐	☐	☐	☐

**3. Over the last 2 weeks how often have you been bothered by any of the following problems?** *(Use "✓" to indicate your answer)*				
	**Not at all**	**Several days**	**More than half the days**	**Nearly every day**
a) Feeling nervous, anxious or on edge	☐	☐	☐	☐
b) Not being able to stop or control worrying	☐	☐	☐	☐
c) Worrying too much about different things	☐	☐	☐	☐
d) Trouble relaxing	☐	☐	☐	☐
e) Being so restless that it is hard to sit still	☐	☐	☐	☐
f) Becoming easily annoyed or irritable	☐	☐	☐	☐
g) Feeling afraid, as though something awful might happen	☐	☐	☐	☐

**4. We would like to know how you have been feeling in the last few weeks. Please circle a number for each statement indicating how often you feel that way, where 1 is almost never, and 5 is almost all the time.**					
	**Almost never**				**Almost all the time**
a) I can laugh and see the funny side of things	1	2	3	4	5
b) I feel cheerful	1	2	3	4	5
c) I look forward with enjoyment to things	1	2	3	4	5

5. Please answer the following items based on how you feel *right now, that is, at the present moment* using the scale below. Try to answer each item as accurately as possible based on your response to that item alone, without regard to your answers to any previous items.

1	2	3	4	5
Strongly disagree				Strongly agree

a) I am feeling optimistic about life's challenges.	☐
b) Right now, I expect things to work out for the best.	☐
c) I am feeling optimistic about my future.	☐
d) I feel that something good will happen today (in the next 24 hours).	☐
e) The future is looking bright to me.	☐
f) At the moment, I expect more to go right than wrong when it comes to my future.	☐
g) I am expecting things to turn out well.	☐

6. On the scale below, please indicate how much you agree with the following statement:

Ever since I can remember, I have been prone to low moods.

Strongly disagree										Strongly agree
0	1	2	3	4	5	6	7	8	9	10

7. The questions in this scale ask about your feelings and thoughts during the last month. In each case, you will be asked to indicate by circling *how often* you felt or thought a certain way.					
	**Never**	**Almost never**	**Some-times**	**Fairly often**	**Very often**
a) In the last month, how often have you been upset because of something that happened unexpectedly?	0	1	2	3	4
b) In the last month, how often have you felt that you were unable to control the important things in your life?	0	1	2	3	4
c) In the last month, how often have you felt nervous and stressed?	0	1	2	3	4
d) In the last month, how often have you felt confident about your ability to handle your personal problems?	0	1	2	3	4
e) In the last month, how often have you felt that things were going your way?	0	1	2	3	4
f) In the last month, how often have you found that you could not cope with all the things that you had to do?	0	1	2	3	4
g) In the last month, how often have you been able to control irritations in your life?	0	1	2	3	4
h) In the last month, how often have you felt that you were on top of things?	0	1	2	3	4
i) In the last month, how often have you been angered because of things that happened that were outside of your control?	0	1	2	3	4
j) In the last month, how often have you felt difficulties were piling up so high that you could not overcome them?	0	1	2	3	4

Section D – Questions about physical health

Choose one option for each questionnaire item.

1. In general, would you say your health is:

Excellent	☐
Very good	☐
Good	☐
Fair	☐
Poor	☐

2. Compared to one year ago, how would you rate your health in general **now**?

Much better now than one year ago	☐
Somewhat better now than one year ago	☐
About the same	☐
Somewhat worse now than one year ago	☐
Much worse now than one year ago	☐

3. The following items are about activities you might do during a typical day. Does **your health now limit** you in these activities? If so, how much?

	**Yes, limited a lot**	**Yes, limited a little**	**No, not limited at all**
a) **Vigorous activities,** such as running, lifting heavy objects, participating in strenuous sports	☐	☐	☐
b) **Moderate activities,** such as moving a table, pushing a vacuum cleaner, bowling, or playing golf	☐	☐	☐
c) Lifting or carrying groceries	☐	☐	☐
d) Climbing **several** flights of stairs	☐	☐	☐
e) Climbing **one** flight of stairs	☐	☐	☐
f) Bending, kneeling, or stooping	☐	☐	☐
g) Walking **more than a mile**	☐	☐	☐
h) Walking **several blocks**	☐	☐	☐
i) Walking **one block**	☐	☐	☐
j) Bathing or dressing yourself	☐	☐	☐

**4.** During the **past 4 weeks,** have you had any of the following problems with your work or other regular daily activities **as a result of your physical health**?		
	**Yes**	**No**
a) Cut down the **amount of time** you spent on work or other activities	☐	☐
b) **Accomplished less** than you would like	☐	☐
c) Were limited in the **kind** of work or other activities	☐	☐
d) Had **difficulty** performing the work or other activities (for example, it took extra effort)	☐	☐

**5.** How much **bodily** pain have you had during the **past 4 weeks?**	
None	☐
Very mild	☐
Mild	☐
Moderate	☐
Severe	☐
Very severe	☐

**6.** During the **past 4 weeks**, how much did **pain** interfere with your normal work (including both work outside the home and housework?	
Not at all	☐
A little bit	☐
Moderately	☐
Quite a bit	☐
Extremely	☐

7. How TRUE or FALSE are **each** of the following statements for you?

	**Definitely true**	**Mostly true**	**Don't know**	**Mostly false**	**Definitely false**
a) I seem to get sick a little easier than other people	☐	☐	☐	☐	☐
b) I am as healthy as anybody I know	☐	☐	☐	☐	☐
c) I expect my health to get worse	☐	☐	☐	☐	☐
d) My health is excellent	☐	☐	☐	☐	☐

Section E – Questions about musculoskeletal pain

Please still answer these questions even if you do not have chronic musculoskeletal pain e.g. 0, no pain

1. Please rate your pain by circling the number that best describes your pain intensity at its **worst** in the last two weeks.

No pain										Pain as bad as you can imagine
0	1	2	3	4	5	6	7	8	9	10

2. Please rate your pain by circling the number that best describes your pain intensity at its **least** in the last two weeks.

No pain										Pain as bad as you can imagine
0	1	2	3	4	5	6	7	8	9	10

3. Please rate your pain by circling the number that best describes your **average** pain intensity over the last two weeks.

No pain										Pain as bad as you can imagine
0	1	2	3	4	5	6	7	8	9	10

4. Please rate your pain by circling the number that best describes how much pain you have **right now**.

No pain										Pain as bad as you can imagine
0	1	2	3	4	5	6	7	8	9	10

5. In the **last two weeks**, how do you feel you have been coping with your pain?

Not coping at all										Coping extremely well
0	1	2	3	4	5	6	7	8	9	10

**6. Circle the number that best describes how, over the past 24 hours, pain has interfered with your:**										
**a) General activity**										
Does not interfere										Pain as bad as you can imagine
0	1	2	3	4	5	6	7	8	9	10
**b) Mood**										
Does not interfere										Pain as bad as you can imagine
0	1	2	3	4	5	6	7	8	9	10
**c) Sleep**										
Does not interfere										Pain as bad as you can imagine
0	1	2	3	4	5	6	7	8	9	10

7. Below are two statements regarding chronic pain. Please rate the truth of each statement as it applies to you. Use the following rating scale to make your choices and circle the number that corresponds with your answer. If you do not have chronic pain, please tick the box stating this.

0	1	2	3	4	5	6
Never true	Very rarely true	Seldom true	Sometimes true	Often true	Almost always true	Always true

a) I lead a full life even though I have chronic pain	0	1	2	3	4	5	6	☐ I don't have chronic pain
b) Before I can make any serious plans, I have to get some control over my pain	0	1	2	3	4	5	6	☐ I don't have chronic pain

Section F – Life events

Below, 12 unpleasant events are listed. Please indicate (by crossing the box) if you have experienced these events in the past 12 months.	
1. You yourself suffered a serious illness, injury, or an assault	☐
2. A serious illness, injury, or assault happened to a close relative	☐
3. Your parent, child or spouse died	☐
4. A close family friend or another relative (aunt, cousin, grandparent) died	☐
5. You had a separation due to marital difficulties	☐
6. You broke off a steady relationship	☐
7. You had a serious problem with a close friend, neighbour, or relative	☐
8. You became unemployed or you were seeking work unsuccessfully for more than 1 month	☐
9. You were sacked from your job	☐
10. You had a major financial crisis	☐
11. You had problems with the police and a court appearance	☐
12. Something you valued was lost or stolen	☐

Section G – Consent to further research
OPTIONAL

There may be further research in this area that follows on from the findings of this study. Please indicate (by crossing the appropriate box) if you are happy to be contacted and invited to participate in other research studies:

I am happy to be contacted about future research	☐
I do not wish to be contacted about future research	☐

Date questionnaire completed:

☐☐ / ☐☐ / 2 0 2 2

THANK YOU

Thank you for completing this questionnaire. Please check you have answered all the questions and return this in the freepost envelope provided.

Please make sure that you also return the consent form with the questionnaire.

If you have any queries or concerns, please contact the De-STRESS pain study team at the University of Southampton at destress@soton.ac.uk.
